# Efficacy of psychological interventions for adolescents with borderline personality disorder: a systematic review and meta-analysis

**DOI:** 10.3389/fpsyt.2026.1833555

**Published:** 2026-06-11

**Authors:** Manqi Cai, Xinxin Song, Jun Tong

**Affiliations:** 1 Faculty of Health and Wellness, City University of Macau, Macao, Macao SAR, China; 2 Department of Child and Adolescent Rehabilitation, Qingdao Mental Health Center, Qingdao, China

**Keywords:** adolescents, borderline personality disorder, clinical significance, dialectical behavior therapy, emotion regulation, mentalization-based treatment, meta-analysis, psychotherapy

## Abstract

**Objective:**

This meta-analysis evaluated the efficacy of psychological interventions for adolescents with borderline personality disorder (BPD) across multiple outcome domains, including BPD symptom severity, emotion regulation, depressive symptoms, general psychopathology, and quality of life.

**Methods:**

Following PRISMA 2020 guidelines (PROSPERO: CRD420261306339), we searched PubMed, PsycINFO, Embase, and Cochrane CENTRAL from inception through January 2026. RCTs of manualized psychological therapies for adolescents aged 12–18 years with BPD or significant BPD features were included. Two reviewers independently extracted data and assessed risk of bias using the Cochrane RoB 2.0 tool. Random-effects meta-analyses were conducted using Hedges’ *g*, with *I*² for heterogeneity and Egger’s test for publication bias.

**Results:**

Twelve RCTs comprising 844 adolescents (mean age = 15.8 years; 81% female) were included. Interventions included DBT-A, MBT-A, CAT, ERT, UP-A, and school-based programs. Psychological interventions produced small significant effects on BPD symptom severity (*k* = 8; *n* = 636; SMD=−0.27; 95% CI [−0.47, −0.06]; *p* = 0.012; *I*²=25%), emotion regulation difficulties (*k* = 5; *n* = 304; SMD=−0.26; 95% CI [−0.48, −0.04]; *p* = 0.020; *I*²=0%), and general psychopathology (*k* = 6; *n* = 302; SMD=−0.34; 95% CI [−0.56, −0.12]; *p* = 0.003; *I*²=0%). No significant effects were observed for depressive symptoms (*k* = 7; *n* = 479; SMD=−0.13; 95% CI [−0.35, 0.09]; *p* = 0.240) or quality of life (*k* = 2; *n* = 126; SMD = 0.05; 95% CI [−0.33, 0.43]; *p* = 0.800). Heterogeneity was low across all outcomes (*I*²=0–29%), and Egger’s test indicated no significant publication bias (all *p*>0.15). Exploratory subgroup analyses by intervention type were limited by single-study subgroups and could not reliably establish differential efficacy. Available follow-up data suggested progressive erosion of treatment gains over 12–36 months.

**Conclusion:**

Psychological interventions for adolescent BPD produced statistically reliable but small improvements in BPD symptoms and emotion regulation, with effect magnitudes that likely fall below clinically meaningful thresholds. No benefits were detected for depressive symptoms or quality of life. Current treatments should be viewed as beneficial but limited. Future research should prioritize adequately powered head-to-head trials, longer follow-up with maintenance interventions, and development of treatments with broader impact across outcome domains.

**Systematic review registration:**

https://www.crd.york.ac.uk/PROSPERO/, identifier CRD420261306339.

## Introduction

### Background and clinical significance

Borderline personality disorder (BPD) in adolescence is associated with substantial clinical complexity. Affected adolescents typically exhibit marked difficulties in emotion regulation, unstable interpersonal relationships, disturbances in identity, and elevated impulsivity (1). In community samples, the prevalence of BPD among adolescents is estimated to be approximately 1–3% (2), whereas rates are substantially higher in clinical and psychiatric settings, where up to one-third of patients may meet diagnostic criteria (3). Given that adolescence represents a period of ongoing neurobiological and personality development, this stage may constitute a particularly important window for early intervention (4, 5).

It is important to acknowledge at the outset that there is no settled consensus on the definition of BPD in either adults or adolescents. Diagnostic criteria differ across systems (DSM-IV, DSM-5 Section II, the DSM-5 Alternative Model for Personality Disorders, and ICD-11), and conceptual frameworks vary along categorical, dimensional, and trait-based lines, with the result that the patient populations described in different trials are often not directly comparable. This clinical heterogeneity is further amplified by very high rates of psychiatric comorbidity in BPD samples at all ages — a feature that is particularly pronounced in adolescence. The eligibility criteria of the present review were designed to accommodate this definitional diversity (see Methods, Eligibility Criteria), while each included trial’s operationalization of BPD is transparently documented in **Table 1**.

**Table 1:** Characteristics of included studies (*k* = 12, *N* = 844).

Study	Country	N	Age	Female	BPD diagnosis	Intervention	Duration	Control	Outcomes
6	Norway	77	15.6 ± 1.5	88.3%	≥2 DSM-IV criteria; 26% full BPD	DBT-A	19 weeks	EUC (weekly psychodynamic/CBT)	Self-harm, suicidal ideation, depression, BPD symptoms
7	Australia	78	16.4 ± 0.9	76%	2–9 DSM-IV criteria; 39% full BPD	CAT	24 sessions	GCC (manualized good clinical care)	BPD symptoms, externalizing/internalizing, functioning
8	Netherlands	43	16.1 ± 1.2	88.4%	≥2 DSM-IV criteria	ERT + TAU	17 weeks + 2 boosters	TAU (naturalistic)	BPD symptoms, emotion dysregulation
9	Netherlands	109	16.0 ± 1.2	96%	≥2 criteria; 73% full BPD	ERT + TAU	17 weeks + 2 boosters	TAU (individual + family)	BPD symptoms, general psychopathology, QoL, emotion dysregulation
10	UK	80	14.7	85%	≥5 criteria (73%)	MBT-A	12 months	TAU (non-manualized)	Self-harm, depression, BPD features
11	Denmark	112	15.8 ± 1.1	99.1%	≥4 criteria; 94.6% full BPD	MBT-G	12 months	TAU (monthly individual)	BPD features, self-harm, depression, functioning
12	Germany	66	16.5 (14-19)	68.2%	100% full BPD diagnosis	PDT (inpatient)	6 months	WL/TAU	Remission, general psychopathology
13	Australia	16	18.4 ± 2.9	81.2%	≥4 criteria; 75% full BPD	HYPE + SFET	16 weeks	SFET (first-episode psychosis)	Depression, functioning, aggression
14	China	20	Adolescents	NR	PDQ-4+ ≥ 5	Meaning-in-life counseling	6 sessions	Waitlist	BPD features, meaning in life
15	UK	32	13-18	75%	BPFSC ≥ 34; 61% ≥5 criteria	BEST (Brief Education Supported Treatment)+ TAU	6 sessions/12 weeks	TAU (school pastoral support)	BPD features, emotion dysregulation, self-harm, QoL
16	Iran	91	15.7 ± 1.0	67%	DSM-5 BPD (SCID-5-PD)	UP-A vs MBT-A	UP-A: 12 wks; MBT-A: 12 mo	Active comparison (MBT-A)	BPD severity, emotion dysregulation, self-harm, impulsivity
17	Germany	120	15.5 ± 1.4	87%	≥2 DSM criteria	TFP-A	12 months	Control condition	Depression, internalizing/externalizing

BPD, borderline personality disorder; DBT-A, Dialectical Behavior Therapy for Adolescents; CAT, Cognitive Analytic Therapy; ERT, Emotion Regulation Training; MBT-A, Mentalization-Based Treatment for Adolescents; MBT-G, group MBT; PDT, Psychodynamic Therapy; HYPE, Helping Young People Early; SFET, Specialist First Episode of Psychosis Treatment; BEST, Brief Education Supported Treatment; UP-A, Unified Protocol for Adolescents; TFP-A, Transference-Focused Psychotherapy for Adolescents; TAU, treatment as usual; EUC, enhanced usual care; GCC, good clinical care; WL, waitlist; NR, not reported; QoL, quality of life.

The functional and clinical consequences of adolescent BPD are considerable. Academic functioning is frequently disrupted, peer relationships are strained, and family conflict is common (18). Rates of self-harm are high, with approximately 70–80% of adolescents engaging in behaviors such as cutting or burning, and lifetime rates of suicide attempts estimated at 60–75% (19). Comorbid conditions, including depressive disorders, trauma-related disorders, and substance use disorders, are also prevalent (20). In the absence of effective intervention, these difficulties often persist into adulthood, contributing to chronic mood symptoms, recurrent interpersonal difficulties, and impaired occupational functioning (21).

### Psychological interventions for adolescent BPD

Among psychological treatments available for adolescents with borderline personality disorder, Dialectical Behavior Therapy for Adolescents (DBT-A) is the most extensively studied approach. DBT-A is specifically adapted from Linehan’s original model (22) for use with adolescents (23) and integrates cognitive–behavioral strategies with mindfulness and distress tolerance skills. The intervention is delivered through multiple treatment modalities, including individual therapy, family-based skills groups, telephone coaching, and therapist consultation teams.

In contrast, Mentalization-Based Therapy for Adolescents (MBT-A) emphasizes attachment processes and the development of mentalizing capacities, with the aim of enhancing adolescents’ ability to understand their own mental states and those of others (10). Additional treatment models include Cognitive Analytic Therapy for Adolescents (CAT; 7), emotion regulation training (9), and adaptations of the Unified Protocol for Adolescents (16). These approaches are grounded in distinct theoretical frameworks and target different therapeutic processes, and early trials suggest potential benefits across models. Previous systematic reviews and meta-analyses have examined aspects of this evidence base (24–26), but have yielded inconsistent conclusions regarding treatment efficacy, differed in scope and inclusion criteria, and have not incorporated the most recent trials.

### Evidence from adult BPD psychotherapy trials

In adults with BPD, the evidence base for psychotherapy is substantially more developed and provides a reference point for the adolescent findings. Cristea et al. (27) conducted a meta-analysis of 33 RCTs with 2,256 participants. They reported a small-to-moderate combined effect of psychotherapy on borderline-relevant outcomes (g around 0.32) at post-treatment, with effects attenuating at follow-up and sustained gains observed primarily for dialectical behavior therapy and psychodynamic approaches. More recent syntheses have reinforced this picture. Stoffers-Winterling et al. (26) confirmed modest but reliable effects of DBT and mentalization-based therapies on self-harm and BPD severity in adults, and Crotty et al. (28), in an AHRQ-commissioned review of 36 trials, concluded that psychotherapies produce small improvements in adult BPD symptoms and self-harm with low-to-moderate certainty of evidence. Three observations across these reviews are relevant to the adolescent literature. First, the pooled effect of structured psychological interventions in adults is small-to-moderate rather than large. Second, effects attenuate over follow-up periods. Third, different therapeutic modalities (DBT, MBT, psychodynamic, transdiagnostic) appear broadly comparable when directly contrasted. Whether the same pattern obtains in adolescents remains an empirical question. Adolescents are a developmentally distinct population in whom personality pathology is more plastic, comorbidity profiles differ, and family and school context play a larger role.

### Study aims

The present systematic review and meta-analysis had three *a priori* aims. The primary aim was to evaluate the efficacy of structured psychological interventions for adolescents with BPD or clinically significant BPD features on BPD symptom severity. Secondary aims were to examine treatment effects on emotion regulation difficulties, depressive symptoms, general psychopathology, and health-related quality of life. An additional aim was to characterize treatment completion and dropout rates and to evaluate the maintenance of treatment effects on BPD symptom severity at the longest available follow-up assessment (≥ 6 months post-treatment). Eligible outcome measures for each domain are described in the Outcome Definitions and Measurement Instruments section. Exploratory subgroup analyses by intervention type were planned *a priori*; however, these analyses were interpreted cautiously given the limited number of studies within each intervention category.

## Methods

This systematic review and meta-analysis was conducted in accordance with the Preferred Reporting Items for Systematic Reviews and Meta-Analyses (PRISMA) 2020 guidelines (29) and the Cochrane Handbook for Systematic Reviews of Interventions (30). The review protocol was prospectively registered on the International Prospective Register of Systematic Reviews (PROSPERO; registration number: CRD420261306339).

### Search strategy

A comprehensive search was conducted across PubMed, PsycINFO, Embase, and Cochrane CENTRAL from database inception through January 10, 2026, with no language restrictions. Search terms covered BPD keywords, adolescent terms, therapy types, and RCT filters (see **Supplementary Table 2** for the full strategy). We also searched ProQuest Dissertations & Theses, ISSPD conference proceedings, and reference lists of included papers and prior reviews.

### Eligibility criteria

Studies were eligible if they met all five criteria. (1) Design: parallel-group or cluster-randomized RCT, including pilot/feasibility trials with outcome data. (2) Population: adolescents aged 12–18 years (or sample mean within range, >75% in band) with either a full DSM-IV/5 BPD diagnosis on a structured interview (SCID-II, SCID-5-PD, CI-BPD, or equivalent) or clinically significant subthreshold features [≥2 DSM-IV/5 criteria on structured interview, or a score at/above the published cutoff on a validated screening instrument, e.g., Borderline Personality Features Scale for Children (BPFSC) ≥34; Personality Diagnostic Questionnaire–4+ (PDQ-4+) BPD subscale ≥5; Personality Assessment Inventory–Borderline Features Scale (PAI-BOR) ≥38]; studies relying solely on unstructured clinical judgment were excluded. (3) Intervention: a manualized psychological intervention, defined as a structured psychotherapy delivered to a published manual, of explicit theoretical orientation, and administered by trained professionals with supervision or fidelity monitoring. Both BPD-specific (DBT-A, MBT-A, CAT, TFP-A, ERT) and transdiagnostic protocols (UP-A, BEST) were eligible. (4) Comparator: any control condition (waitlist, TAU, active psychological, or pharmacological); pharmacotherapy-only arms were excluded. In multi-arm trials, data from the eligible psychological arm and the most comparable non-psychological control arm were extracted. (5) Outcomes: quantitative data on at least one pre-specified outcome domain (see Outcome Definitions, below).

### Outcome definitions and measurement instruments

Outcomes were defined *a priori* in the PROSPERO protocol and operationalized below. For each outcome, a closed list of eligible instruments was specified, and effect sizes were extracted only from instruments on that list.

#### Primary outcome: BPD symptom severity

Defined as the total severity of borderline personality features measured by validated, disorder-specific instruments: the Borderline Symptom List–23 (BSL-23; 31), the Borderline Personality Features Scale for Children (BPFSC/BPFSC-11; 32), the Zanarini Rating Scale for BPD (ZAN-BPD; 33), the Childhood Interview for DSM-IV BPD (CI-BPD), or the SCID-II/SCID-5-PD BPD dimensional score. Where multiple eligible instruments were reported, the instrument-selection hierarchy described in the Data Extraction section was applied.

#### Secondary outcome 1: emotion regulation difficulties

Defined as the degree of impairment in identifying, accepting, and modulating emotional responses, measured primarily by the Difficulties in Emotion Regulation Scale (DERS; 34) or the Emotion Regulation Questionnaire (ERQ; 35). Where a trial did not administer a dedicated ER instrument but reported an emotion-regulation-relevant subscale of a BPD-specific instrument (e.g., affective instability subscales of the BPFSC or BSL), this subscale was used as a fallback measure; the lower construct specificity of these fallback measures is acknowledged in the interpretation of pooled effects. Total scores were used for dedicated instruments; where only subscale data were available, the most theoretically aligned subscale was extracted and reported.

#### Secondary outcome 2: depressive symptoms

Defined as the severity of depressive symptomatology over a recent reference period (typically two weeks), measured by validated depression scales: the Beck Depression Inventory–II (BDI-II), the Mood and Feelings Questionnaire or its short form (MFQ/SMFQ), or the Montgomery–Åsberg Depression Rating Scale (MADRS). Clinician-rated instruments (e.g., MADRS) were preferred over self-report when both were available.

#### Secondary outcome 3: general psychopathology

Defined as overall non-specific symptom burden across internalizing and externalizing domains, measured by broad-bandwidth instruments: the Youth Self-Report (YSR), the Child Behavior Checklist (CBCL), the Symptom Checklist–90–Revised (SCL-90-R) Global Severity Index, the Brief Symptom Inventory (BSI), or the Strengths and Difficulties Questionnaire (SDQ) total score. This construct is distinct from BPD symptom severity: BPD severity captures personality-pathology-specific features (identity disturbance, affective instability, interpersonal disturbance, impulsivity, self-harm), whereas general psychopathology indexes co-occurring symptomatology that is not specific to personality pathology (e.g., anxiety, somatic complaints, conduct problems). The two constructs may move in different directions in response to BPD-targeted treatment.

#### Secondary outcome 4: health-related quality of life

Defined as self- or proxy-reported functioning and well-being across multiple life domains (physical, psychological, social, environmental/school), measured by validated multi-domain QoL instruments (e.g., EQ-5D-5L, WHOQOL-BREF, KIDSCREEN-27/52, PedsQL). The two contributing trials used the EQ-5D-5L (15) and a multi-domain self-report QoL instrument as reported in the original trial (9). For Wilson et al. (15), QoL data were available for the 12-week post-randomization sub-sample only (*n* = 17 of 32 randomized; the remaining participants had not reached the 12-week assessment timepoint when the trial was suspended due to COVID-19); the pooled QoL sample (*n* = 126) therefore comprises Schuppert et al. (9; *n* = 109) and the Wilson et al., 12-week sub-sample (*n* = 17). Where only domain-specific scores were available, the total/global score was extracted in preference to subscales.

#### Additional outcomes: treatment completion, dropout, and follow-up maintenance

Treatment completion was defined as the proportion of randomized participants completing the assigned intervention protocol per the original trial’s definition (typically attendance at a pre-specified minimum number of sessions). Dropout was defined as the proportion of randomized participants who discontinued the intervention or were lost to assessment before the post-treatment timepoint, with reasons extracted where reported. Follow-up maintenance was assessed as the standardized mean difference on the primary outcome of BPD symptom severity (using the same instrument as in the post-treatment analysis for each trial) at the longest available follow-up timepoint ≥6 months post-treatment. Follow-up outcomes and the maintenance of treatment effects were explicitly pre-specified in the PROSPERO protocol; treatment completion and dropout were not separately listed in the registered protocol but were tracked from the outset of data extraction as descriptors of trial feasibility, and this is acknowledged here in line with the PROSPERO statement that protocol deviations would be transparently reported. Certainty of evidence assessment using the GRADE framework was registered in the PROSPERO protocol; however, given the small number of trials per outcome (*k* = 2–8) and the resulting imprecision affecting every GRADE domain uniformly, a formal GRADE rating was judged uninformative for this evidence base. We therefore report risk-of-bias judgments per RoB 2 at the trial level and discuss limitations narratively in the Discussion. This represents a deviation from the registered protocol and is disclosed in accordance with PROSPERO’s requirement of transparent reporting of protocol deviations.

### Risk of bias assessment

Risk of bias for each included trial was assessed independently by two reviewers using the Cochrane Risk of Bias 2.0 tool (RoB 2; 36), which evaluates five domains: (1) bias arising from the randomization process; (2) bias due to deviations from intended interventions (assessed under the intention-to-treat effect of assignment); (3) bias due to missing outcome data; (4) bias in measurement of the outcome; and (5) bias in selection of the reported result. Within each domain, a series of signaling questions was answered and combined according to the RoB 2 algorithm to yield a domain-level judgment of low risk, some concerns, or high risk; an overall risk-of-bias judgment per outcome was derived following the RoB 2 algorithm. Assessments were anchored to the primary outcome of BPD symptom severity, and conducted separately for secondary outcomes where the rating differed (e.g., owing to differences in outcome ascertainment between domains). Disagreements between the two reviewers were resolved by discussion and, where unresolved, adjudicated by a third reviewer. Risk-of-bias judgments informed pre-specified sensitivity analyses restricted to trials with an overall judgment of low risk of bias.

### Statistical analysis

#### Data extraction

Two reviewers independently extracted data using a standardized form. Extracted variables included study location, year, and sample size; participant demographics and clinical characteristics; intervention and control condition details; and outcome data at each available timepoint. When studies reported multiple measures of the same construct, a pre-specified instrument-selection hierarchy was applied to reduce construct heterogeneity across pooled effects: (a) disorder-specific instruments with established psychometric properties in adolescent BPD samples (e.g., BSL-23, BPFSC, ZAN-BPD) were prioritized over generic broad-band measures (e.g., SCL-90 borderline subscale); (b) clinician-rated instruments were preferred over self-report when both were available and the construct was identical; and (c) where instruments were of comparable specificity, the measure with the larger published validation evidence in the adolescent age range was selected. The full list of eligible instruments per outcome domain is provided in the Outcome Definitions and Measurement Instruments section above. Both post-treatment and longest available follow-up data were extracted. Disagreements were resolved by a third reviewer.

#### Effect size calculation

Effect sizes were calculated as Hedges’ *g* with 95% confidence intervals, computed from means and standard deviations where available, or converted from F, t, or p values following standard formulas (37). Change scores were used when baseline values differed between groups; otherwise, post-treatment scores were used. Missing standard deviations were imputed from studies with comparable samples and measures. Intention-to-treat data were preferred over completer analyses.

#### Meta-analytic procedures

We used random-effects models with DerSimonian-Laird estimation. For multiple effect sizes from the same study, we applied robust variance estimation with small-sample corrections (38). Heterogeneity was assessed using *I*², interpreted according to conventional thresholds: below 25% as low, around 50% as moderate, and above 75% as high (39). Throughout, *k* denotes the number of effect sizes contributing to a pooled estimate; for outcomes where multiple effect sizes were extracted from the same trial (via robust variance estimation), the number of contributing studies is reported separately in the Results.

#### Publication bias and sensitivity analyses

Publication bias was assessed using funnel plots and Egger’s regression test (threshold *p* <.10). Sensitivity analyses included leave-one-out analysis, restriction to studies rated as low risk of bias, and comparison of samples with full BPD diagnosis versus subthreshold features. Treatment subgroup analyses were planned but remained exploratory given the small number of studies per group. We conducted all analyses in Stata 17.

## Results

### Study selection and characteristics

**Figure 1:**
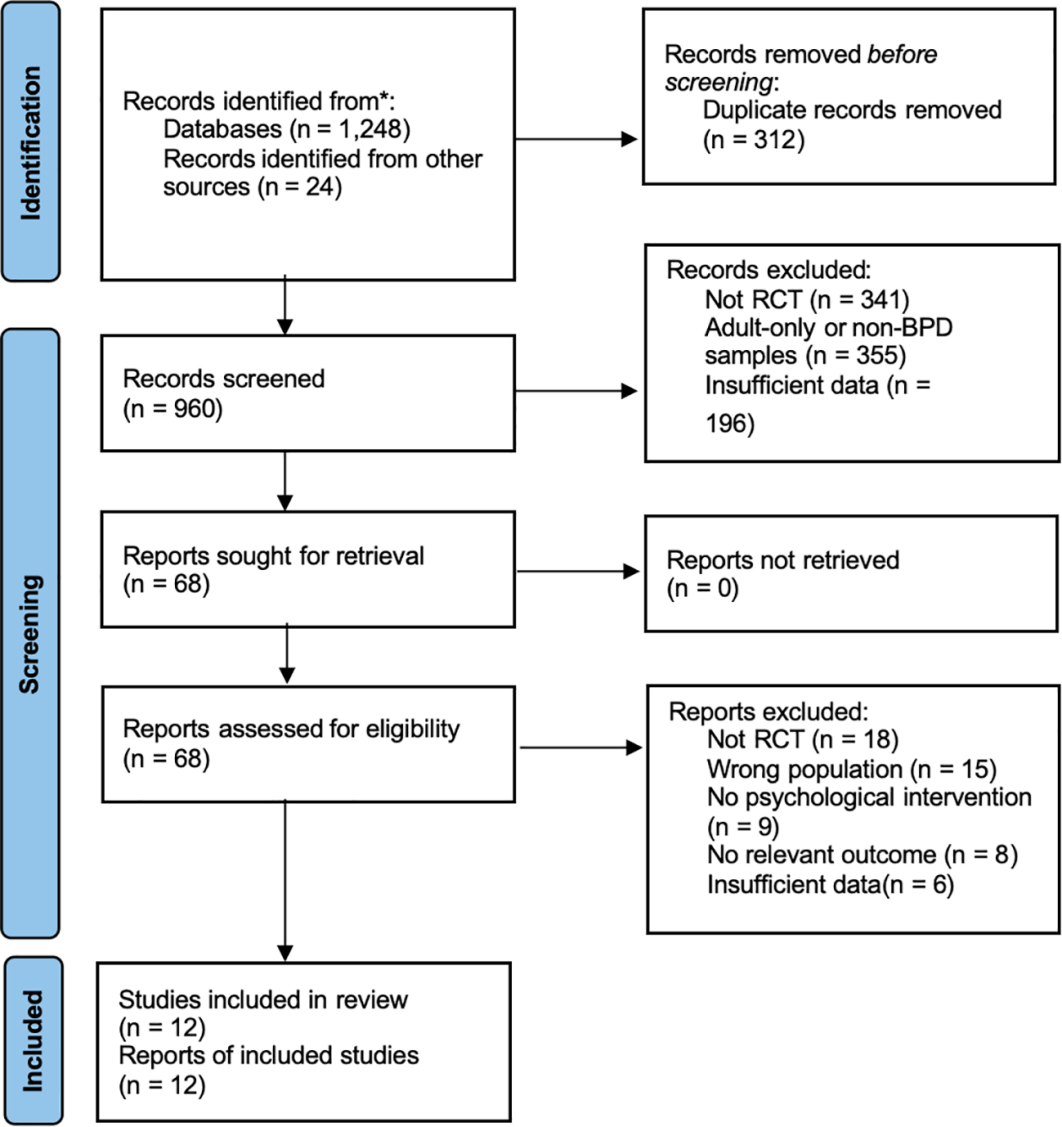
PRISMA flow diagram of study selection. The flow diagram illustrates the identification, screening, eligibility assessment, and inclusion of studies in accordance with PRISMA 2020 guidelines (29).

The database searches identified 1,248 records from databases (with no additional records from trial registers). A further 24 records were identified through supplementary searches of reference lists of included papers and prior reviews, ProQuest Dissertations & Theses, and ISSPD conference proceedings. After removal of 312 duplicate records, 960 unique records were screened by title and abstract by two reviewers independently, of which 892 were excluded at this stage as clearly irrelevant (principal reasons: not RCT, *n* = 341; adult-only or non-BPD samples, *n* = 355; insufficient data, *n* = 196). The remaining 68 reports were sought for retrieval, all of which were retrieved (*n* = 0 reports not retrieved). All 68 full-text reports were assessed against the eligibility criteria, with 56 excluded for the following pre-specified reasons: not RCT (*n* = 18); wrong population (*n* = 15); no psychological intervention (*n* = 9); no relevant outcome (*n* = 8); insufficient data (*n* = 6). Twelve unique RCTs were retained for quantitative synthesis (see **Figure 1**). The total sample comprised 844 participants (range: 16 to 120 per study), with a mean age of 15.8 years (*SD* = 0.9) and 81% female representation; studies were conducted across eight countries. Where numerically reported (4 of 12 studies), the rate of comorbid depressive disorder ranged from 19.8% to 67% (unweighted mean = 42.7%); a fifth study (17) reported 86.6% depressive-episode prevalence at admission but was excluded from this summary because the sample comprised day-clinic patients with admission-defined diagnoses rather than community-defined Axis-I comorbidity. These figures are consistent with the established overlap between BPD and depressive psychopathology in adolescent samples. Per-study comorbid depression rates are as follows: Chanen et al. (7), ~67% (depressive disorder); Rossouw & Fonagy (10), ~56% (depressive disorder); Gleeson et al. (13), ~28% (mood-/depression-related); Mohajerin et al. (16), 19.8% (major depressive disorder); and Jahn et al. (17), 86.6% (depressive episode at admission, day-clinic sample). Comorbid depression was not numerically reported in Mehlum et al. (6) Schuppert et al. (, 8, 9); Beck et al. (11) Li (14); or Salzer et al. (12) Wilson et al. (15) recruited a mixed conduct/emotion disorder sample without separately quantifying depressive comorbidity.

### Intervention duration and frequency by type

**Figure 2:**
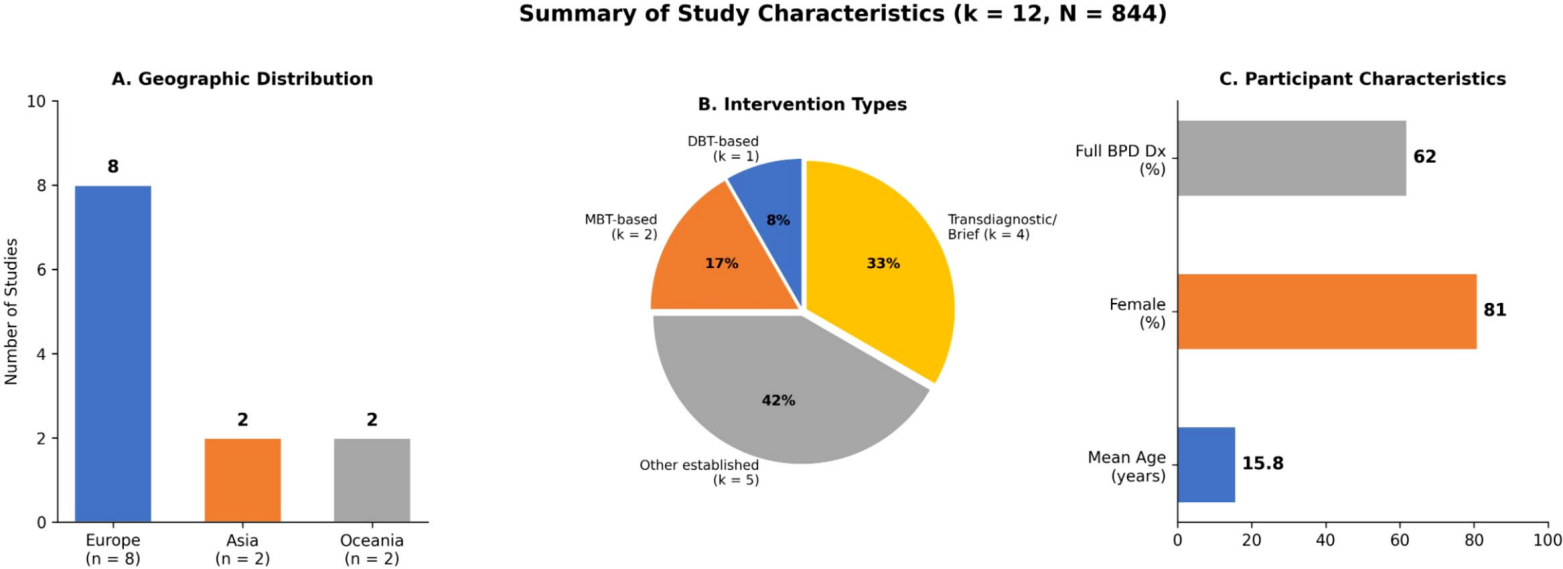
Summary of study characteristics. Panel **(A)** shows the geographic distribution of included studies (*k* = 12): Europe (*n* = 8: Norway, Netherlands ×2, UK ×2, Denmark, Germany ×2), Asia (*n* = 2: China, Iran), Oceania (*n* = 2: Australia ×2). Panel **(B)** displays intervention types: DBT-based (*k* = 1), MBT-based (*k* = 2), other established therapies (CAT, ERT × 2, PDT, TFP-A; *k* = 5), transdiagnostic/brief interventions (*k* = 4). Panel **(C)** summarizes participant characteristics: mean age 15.8 years, 81% female, 62% meeting full BPD diagnostic criteria.

To facilitate interpretation of the heterogeneity in treatment dosage, included interventions are grouped below into the four categories displayed in **Figure 2B** (full per-study details are in **Table 1**). DBT-based (*k* = 1; 6) delivered 19 weeks of combined individual therapy, multifamily skills group, and skills-coaching contact, with a total dose of approximately 80 hours per participant. MBT-based (*k* = 2; 10, 11) delivered 12 months of weekly individual or group mentalization-based sessions, with total session counts of approximately 37–50 sessions per participant. Other established therapies (*k* = 5; CAT, ERT × 2, PDT, TFP-A) ranged from 17 weeks (ERT plus two booster sessions; 8, 9) to 12 months (TFP-A; 17) in total duration, with session frequency of 1–3 per week; CAT (7) delivered 24 sessions, while inpatient PDT (12) comprised approximately 6 months of daily multimodal treatment—the most intensive protocol in the review. Transdiagnostic and brief interventions (*k* = 4; UP-A, BEST, HYPE + SFET, meaning-in-life counselling) were substantially shorter: UP-A delivered 12 weekly sessions (16); BEST delivered 6 sessions over 12 weeks (15); HYPE + SFET delivered 16 weeks of weekly individual sessions for adolescents with co-occurring first-episode psychosis (13); and meaning-in-life counselling delivered 6 weekly group sessions (14). Total treatment dose (estimated as sessions × mean session duration) thus varied by approximately an order of magnitude across the included trials, from ≈6 hours for the briefest transdiagnostic protocol to >100 hours for the longest specialized multimodal protocols. This dose heterogeneity is considered in the interpretation of subgroup analyses and in the Discussion.

### Primary outcome: BPD symptom severity

**Figure 3:**
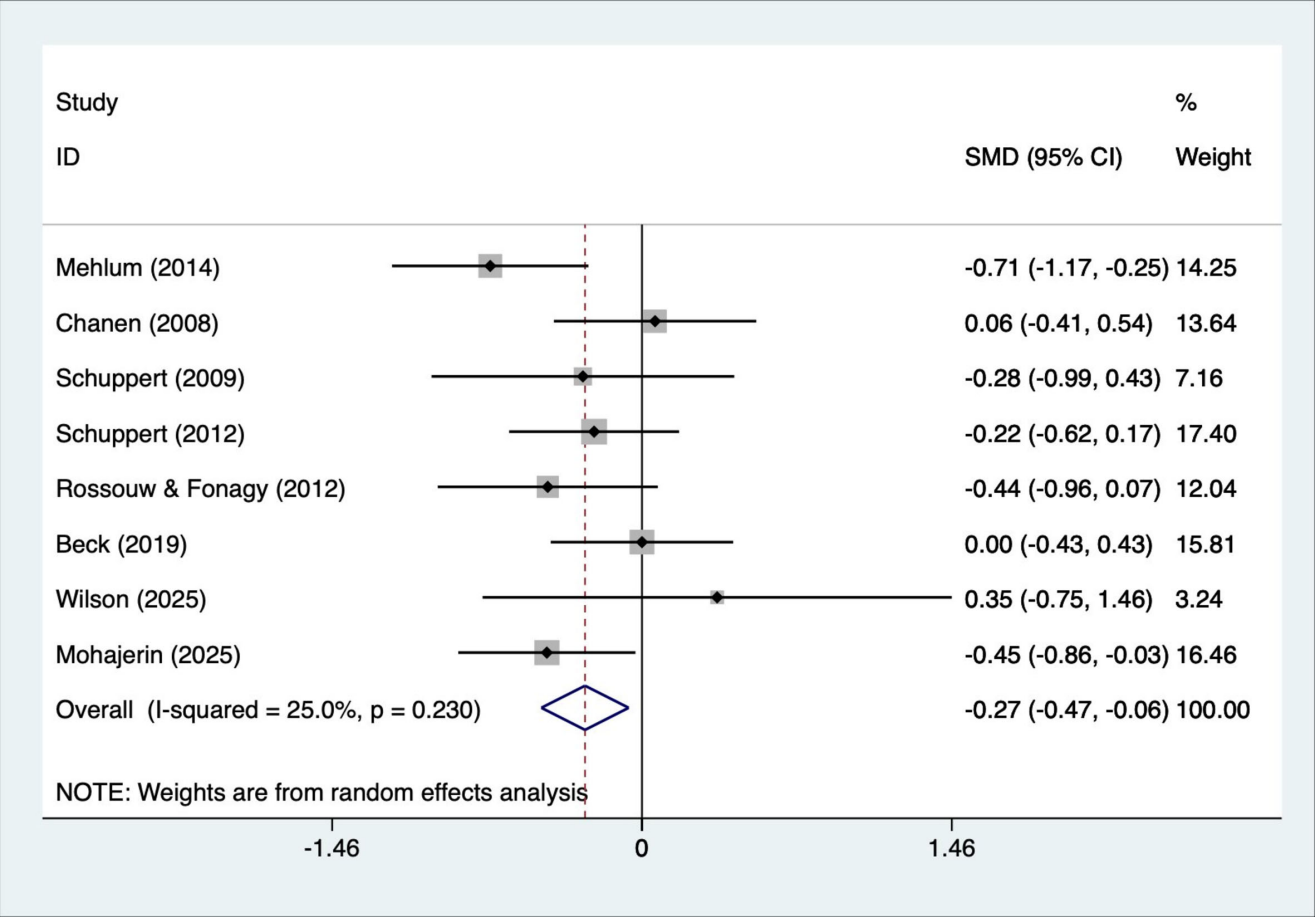
Forest plot: effect of psychological interventions on BPD symptom severity. Random-effects meta-analysis demonstrating a small significant effect favoring interventions (SMD = -0.27, 95% CI: -0.47 to -0.06, *p* = .012). Diamond indicates pooled effect estimate.

**Figure 4:**
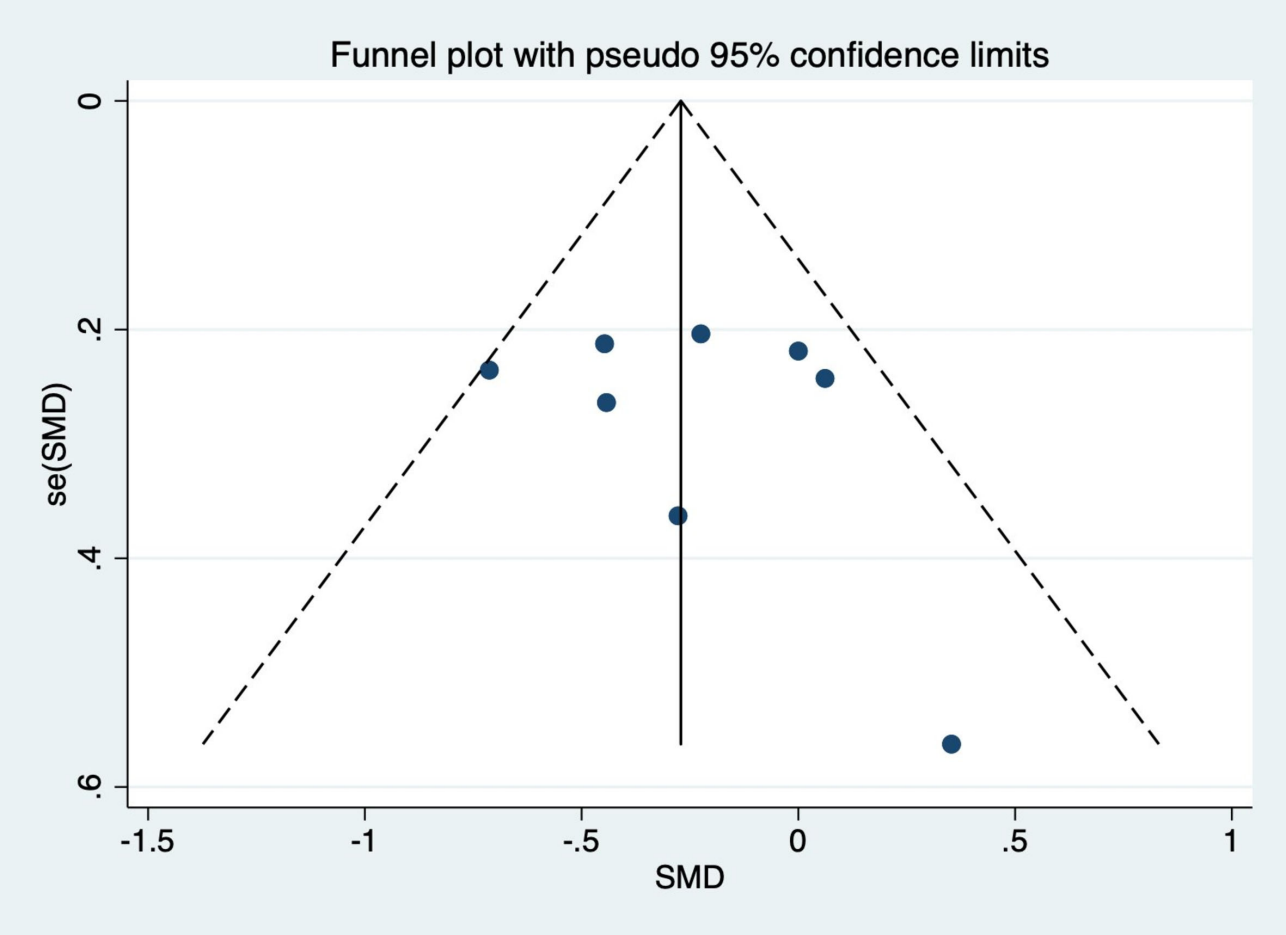
Funnel plot for BPD symptom severity (Egger’s *p* = .531).

Eight studies (*n* = 636 participants) provided data on BPD symptom severity. Random-effects meta-analysis revealed a significant small effect favoring psychological interventions over control conditions (SMD = -0.27, 95% CI: -0.47 to -0.06, *Z* = 2.51, *p* = .012; **Figure 3**). Heterogeneity was low (I^2^ = 25.0%, *p* = .230), suggesting consistent effects across studies. Egger’s test indicated no significant publication bias (*p* = .531) (**Figure 4**).

Exploratory subgroup analysis by intervention type revealed notable variation, though results should be interpreted with caution given the small number of studies per subgroup (*k* = 1-2) (**Table 2**). DBT-A showed a large effect based on a single study (6; SMD = -0.71, 95% CI: -1.17 to -0.25, *n* = 77), followed by UP-A (SMD = -0.45, 95% CI: -0.86 to -0.03) and MBT-A (SMD = -0.44, 95% CI: -0.96 to 0.07). CAT, MBT-G, and BEST showed non-significant effects. Given the single-study basis for most subgroups, differential intervention efficacy cannot be reliably established.

**Table 2:** Exploratory subgroup analyses by intervention type for BPD symptom severity.

Intervention	k	SMD (95% CI)	I^2^	Weight
DBT-A	1	-0.71 (-1.17, -0.25)*	—	14.25%
UP-A	1	-0.45 (-0.86, -0.03)*	—	16.46%
MBT-A	1	-0.44 (-0.96, 0.07)	—	12.04%
ERT + TAU	2	-0.24 (-0.59, 0.11)	0.0%	24.56%
CAT	1	0.06 (-0.41, 0.54)	—	13.64%
MBT-G	1	0.00 (-0.43, 0.43)	—	15.81%
BEST + TAU	1	0.35 (-0.75, 1.46)	—	3.24%
Overall	8	-0.27 (-0.47, -0.06)*	25.0%	100%

*p <.05. DBT-A, Dialectical Behavior Therapy for Adolescents; UP-A, Unified Protocol for Adolescents; MBT-A/G, Mentalization-Based Treatment (individual/group); ERT, Emotion Regulation Training; CAT, Cognitive Analytic Therapy; BEST, Brief Education Supported Treatment; TAU, Treatment as Usual.

### Secondary outcome: emotion regulation difficulties

**Figure 5:**
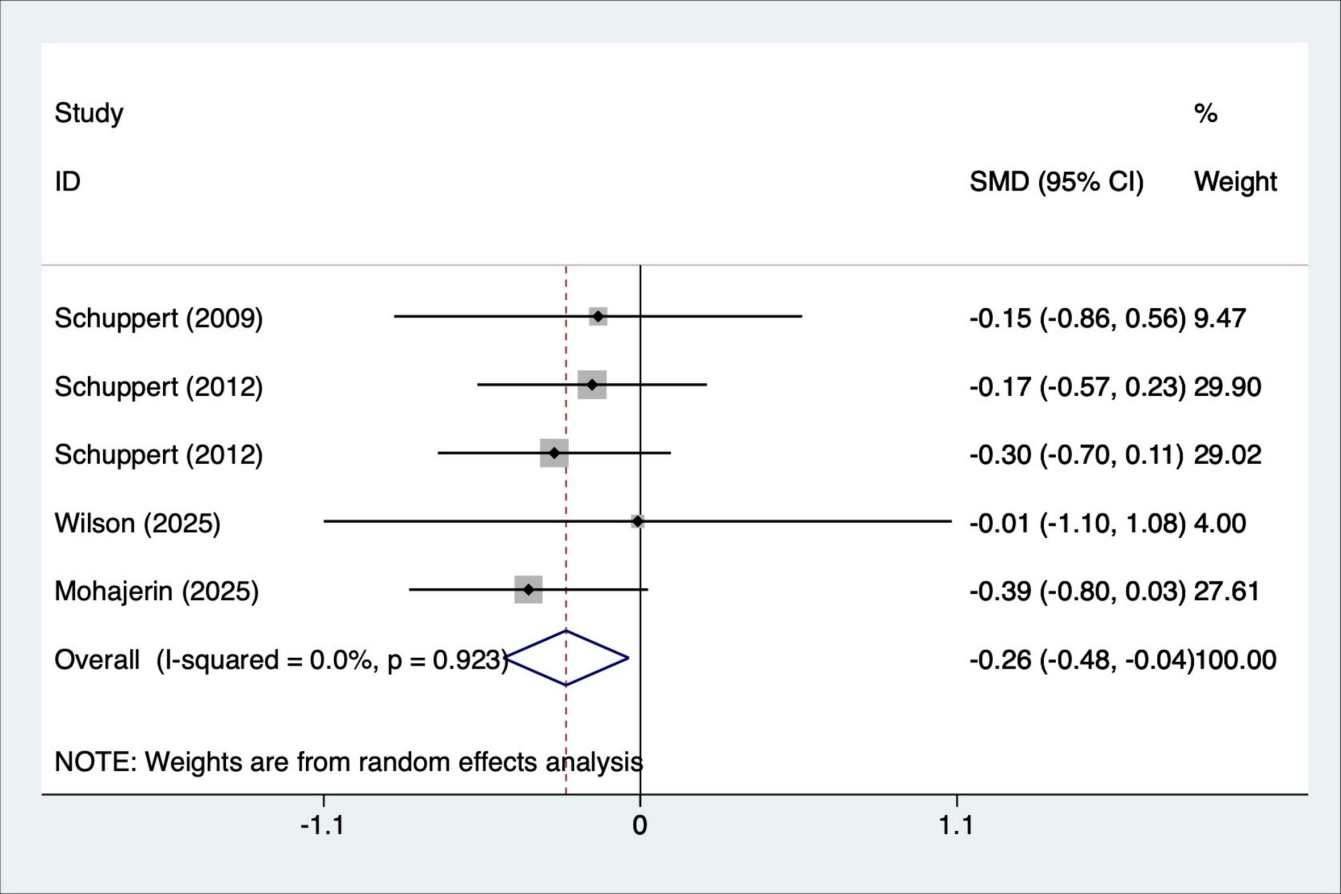
Forest plot: effect on emotion regulation difficulties. SMD = -0.26 (95% CI: -0.48 to -0.04), *p* = .020, *I*² = 0.0%.

**Figure 6:**
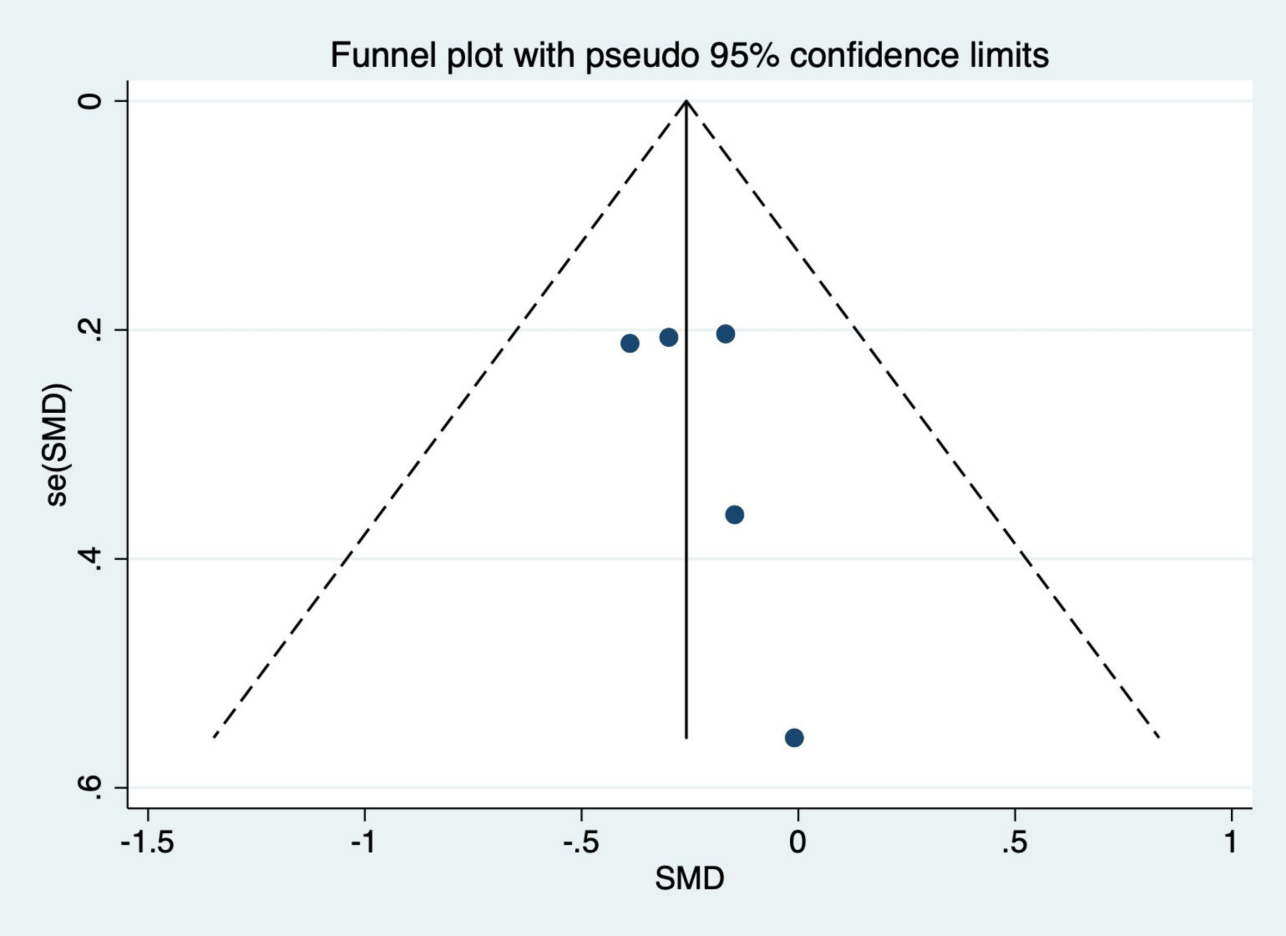
Funnel plot for emotion regulation (Egger’s *p* = .311).

Four studies contributing five effect sizes (*n* = 304) assessed emotion regulation difficulties. Meta-analysis demonstrated a significant small effect favoring psychological interventions (SMD = -0.26, 95% CI: -0.48 to -0.04, *p* = .020; **Figure 5**). Exploratory subgroup analysis by intervention type is presented in **Supplementary Figure 2**. Heterogeneity was negligible (I^2^ = 0.0%) (**Figure 6**).

### Secondary outcome: depressive symptoms

**Figure 7:**
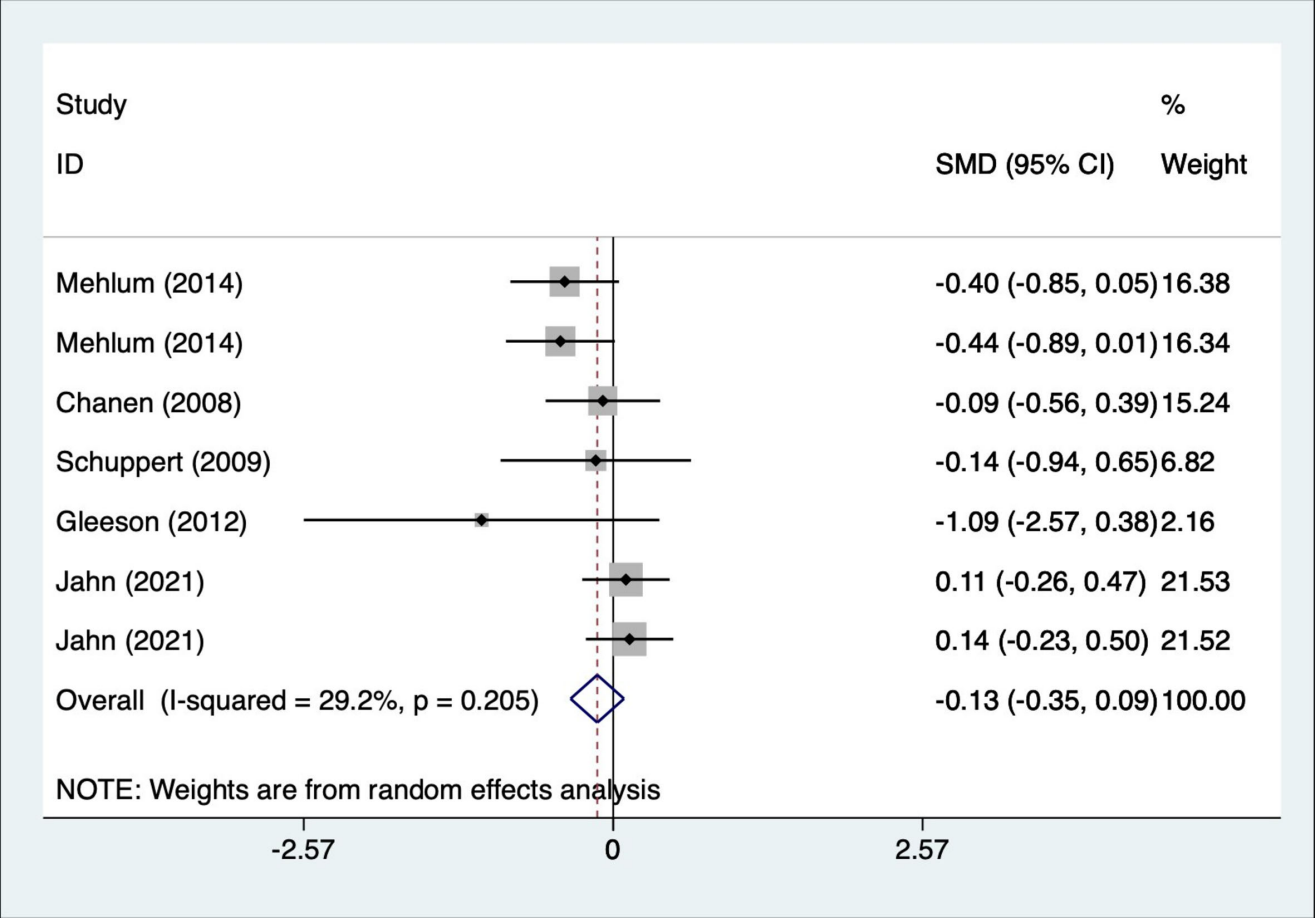
Forest plot: effect on depressive symptoms (non-significant). SMD = -0.13 (95% CI: -0.35 to 0.09), *p* = .24 (non-significant). The 95% CI crosses zero.

**Figure 8:**
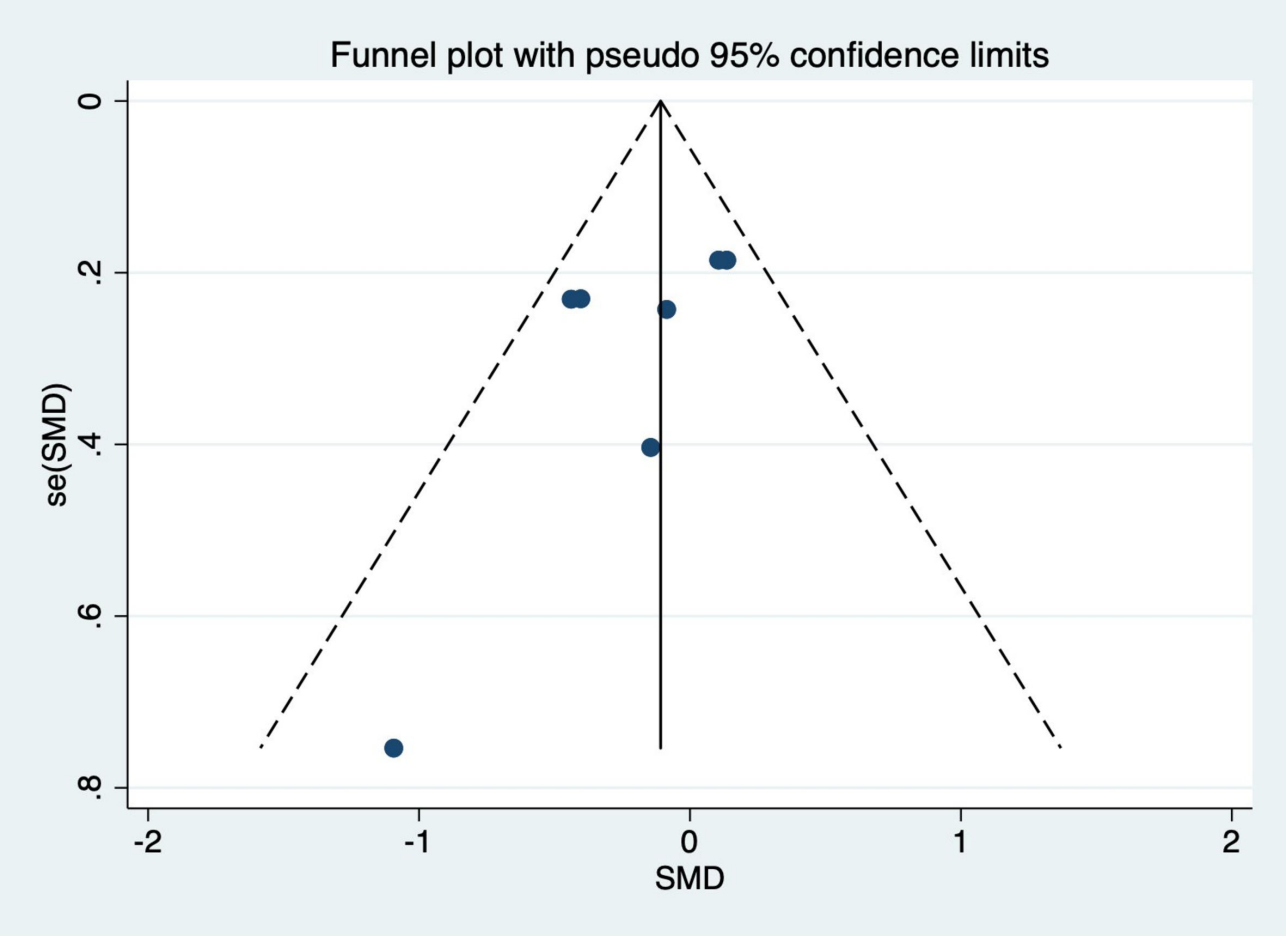
Funnel plot for depressive symptoms (Egger’s *p* = .155).

Five studies contributing seven effect sizes (*n* = 479) reported depressive symptoms. Psychological interventions showed a non-significant effect (SMD = -0.13, 95% CI: -0.35 to 0.09, *p* = .24; **Figure 7**), with low-to-moderate heterogeneity (I^2^ = 29.2%). Exploratory subgroup analysis by intervention type is presented in **Supplementary Figure 1**; the corresponding funnel plot is shown in **Figure 8**.

### Secondary outcome: general psychopathology

**Figure 9:**
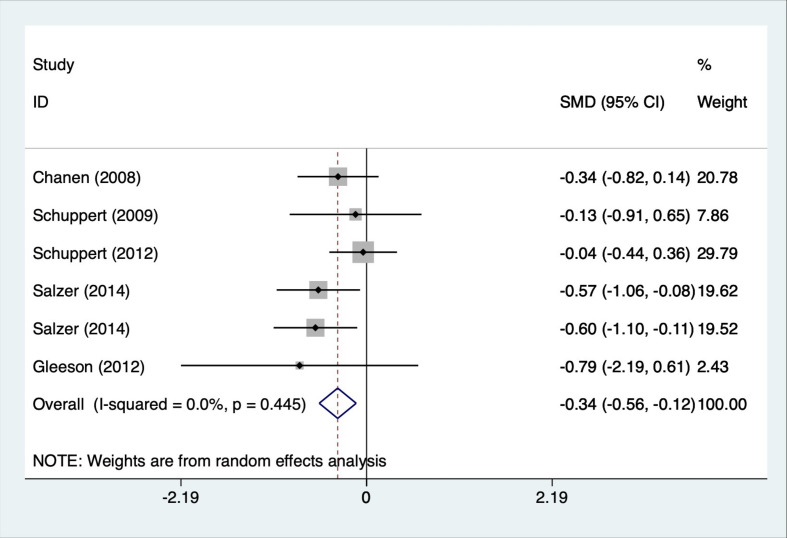
Forest plot: effect on general psychopathology. SMD = -0.34 (95% CI: -0.56 to -0.12), *p* = .003, *I*² = 0.0%.

**Figure 10:**
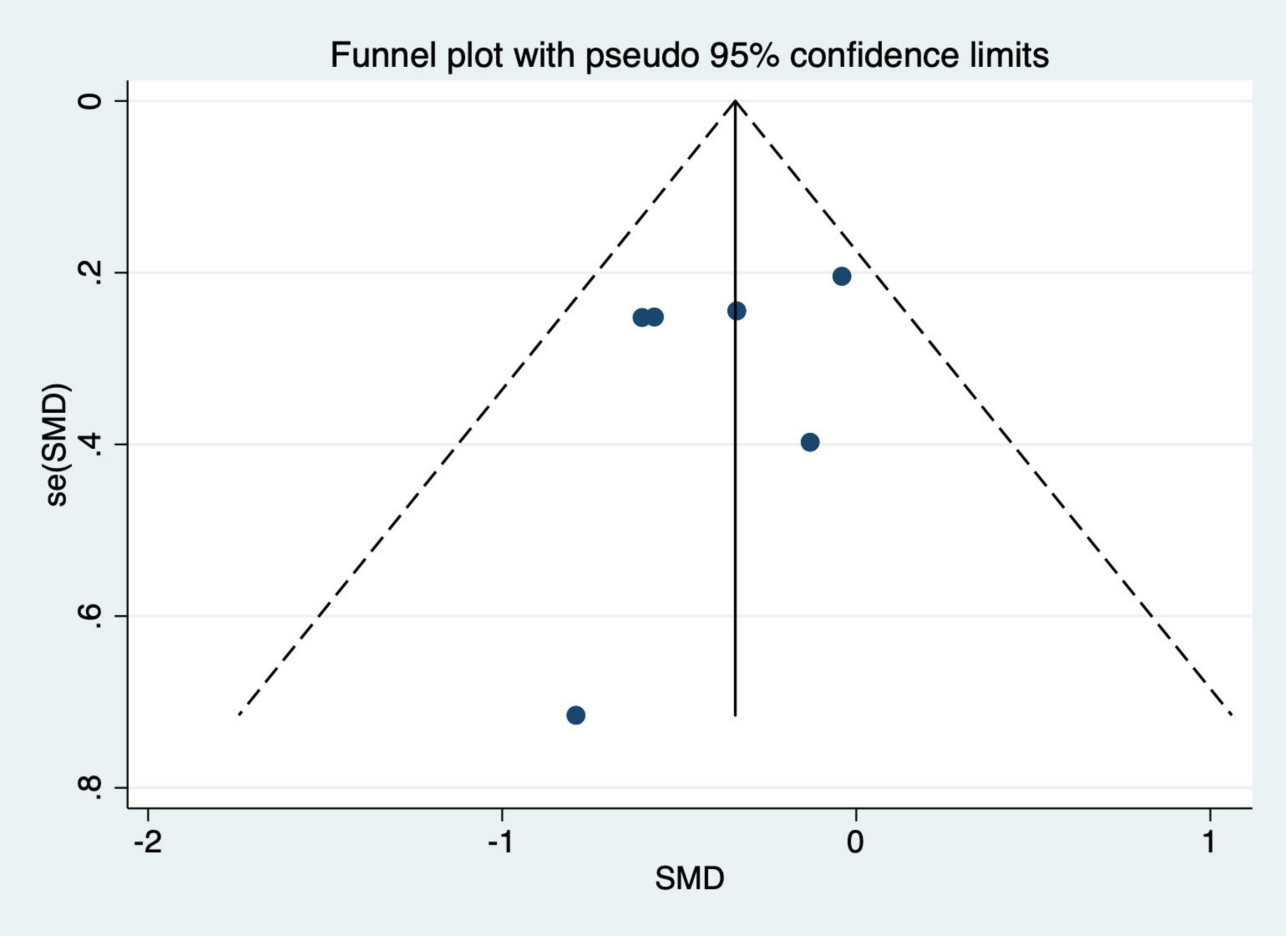
Funnel plot for general psychopathology (Egger’s *p* = .525).

Five studies contributing six effect sizes (*n* = 302) provided data on general psychopathology. Meta-analysis revealed a significant small-to-moderate effect (SMD = -0.34, 95% CI: -0.56 to -0.12, *p* = .003; **Figure 9**). Exploratory subgroup analysis by intervention type is presented in **Supplementary Figure 3**. Heterogeneity was negligible (I^2^ = 0.0%) (**Figure 10**).

### Secondary outcome: quality of life

**Figure 11:**
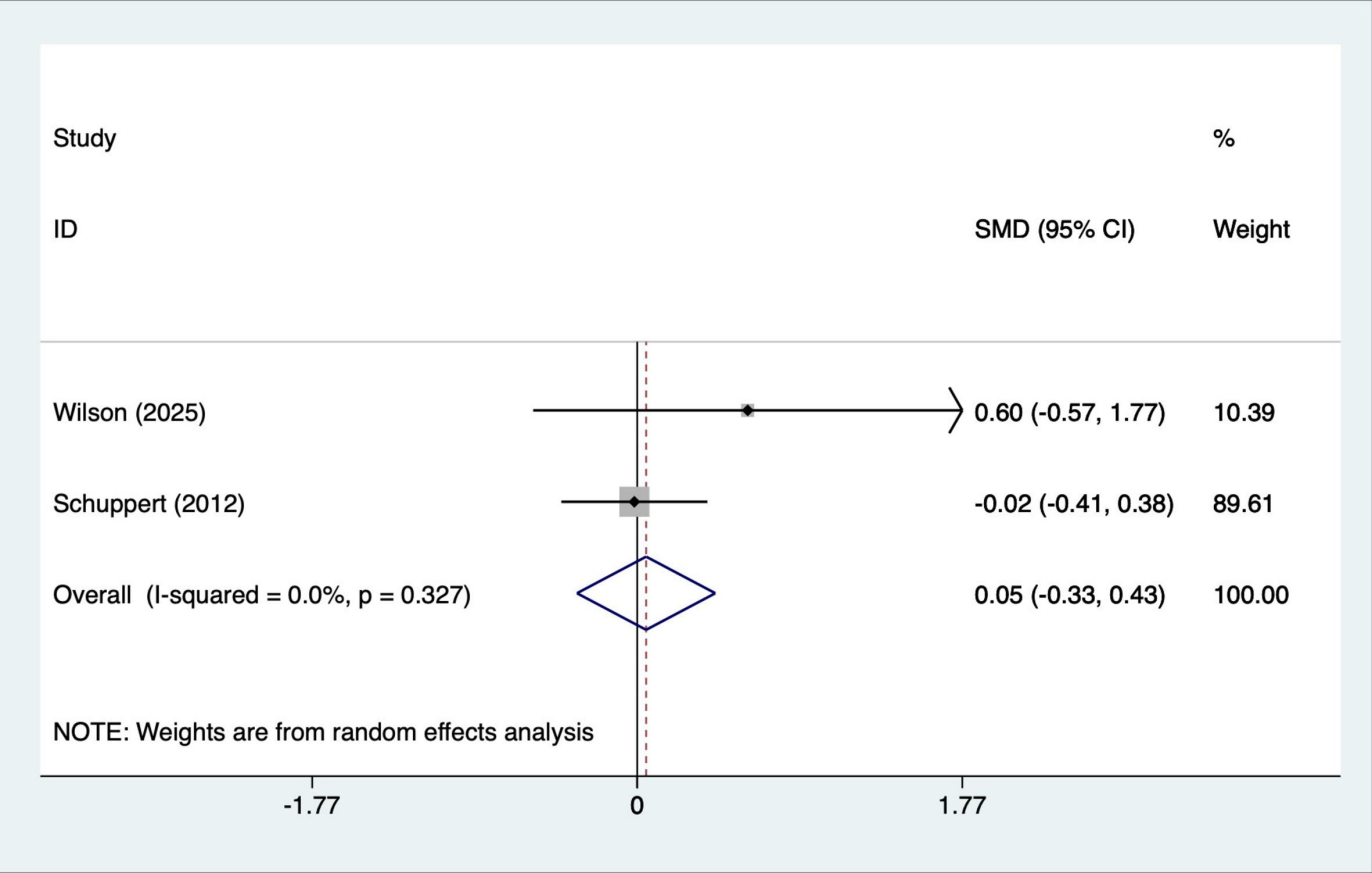
Forest plot: effect on quality of life (non-significant). SMD = 0.05 (95% CI: -0.33 to 0.43), *p* = .80 (non-significant). The effect is essentially zero.

Two studies (*n* = 126) reported quality of life outcomes. Meta-analysis showed a non-significant effect (SMD = 0.05, 95% CI: -0.33 to 0.43, *p* = .80; **Figure 11**).

The results showed a differential pattern across outcome domains: interventions reduced BPD-specific symptoms (SMD = -0.27, *p* = .012) and general psychopathology (SMD = -0.34, *p* = .003) but had no significant effects on depressive symptoms (SMD = -0.13, *p* = .24) or quality of life (SMD = 0.05, *p* = .80) (**Table 3**).

**Table 3:** Summary of meta-analysis results across outcome domains.

Outcome	k	n	SMD (95% CI)	*p*	I^2^	Egger’s *p*
BPD symptom severity	8	636	-0.27 (-0.47, -0.06)	.012*	25.0%	.531
Emotion regulation	5	304	-0.26 (-0.48, -0.04)	.020*	0.0%	.311
Depressive symptoms	7	479	-0.13 (-0.35, 0.09)	.24	29.2%	.155
General psychopathology	6	302	-0.34 (-0.56, -0.12)	.003*	0.0%	.525
Quality of life	2	126	0.05 (-0.33, 0.43)	.80	0.0%	—

*k*, number of effect sizes; *n*, total participants; SMD, standardized mean difference (Hedges’ *g*); CI, confidence interval. *p <.05.

### Treatment completion and dropout

Across all included studies, the mean treatment completion rate was 76.3% (range: 58-92%). The weighted mean dropout rate was 23.7% (*SD* = 11.2%), which is comparable to dropout rates reported in adult BPD treatment trials (20-30%). Dropout rates were typically higher in longer-duration interventions than in briefer protocols. Reasons for dropout, where reported, included loss to follow-up, withdrawal of consent, hospitalization, and adverse events unrelated to treatment. No study reported serious adverse events directly attributable to the psychological intervention.

### Risk of bias assessment

**Figure 12:**
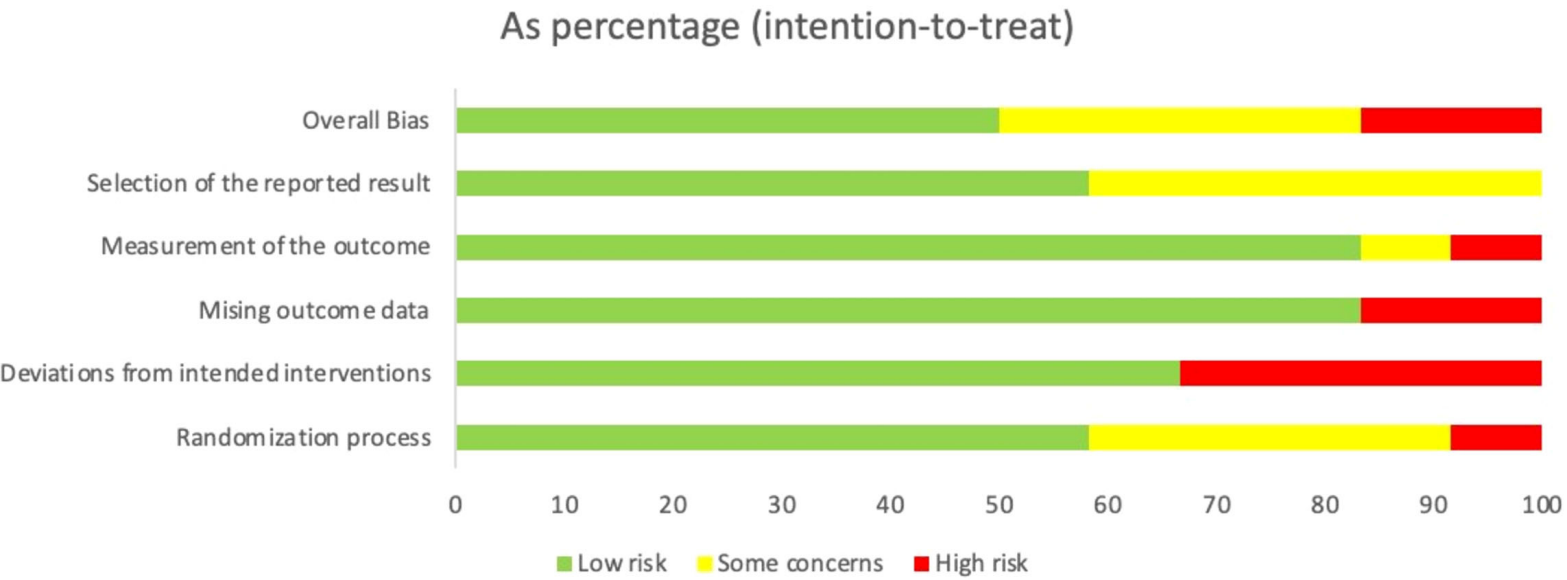
Risk of bias assessment summary (intention-to-treat analysis). Risk of bias summary showing the percentage of studies at low risk (green), with some concerns (yellow), and high risk (red) across each domain. Overall, approximately 50% of studies were judged as low risk of bias, with the most common concerns arising from deviations from intended interventions and selection of the reported result.

Risk of bias was assessed using the Cochrane Risk of Bias tool 2.0 (RoB 2). **Figure 12** presents the summary of risk of bias assessments across all included studies using an intention-to-treat analysis framework. The assessment evaluated five domains: randomization process, deviations from intended interventions, missing outcome data, measurement of the outcome, and selection of the reported result; an overall risk-of-bias judgment was derived per the RoB 2 algorithm, consistent with the procedure described in the Methods.

### Sensitivity analyses

Sensitivity analyses supported the robustness of the primary findings. Leave-one-out analysis for BPD symptom severity showed that no single study substantially influenced the pooled estimate (range of SMD when each study removed: -0.21 to -0.32). When analyses were restricted to studies with low overall risk of bias (*k* = 6), the effect remained significant (SMD = -0.29, 95% CI: -0.52 to -0.06), similar to the main analysis. Comparing studies requiring full BPD diagnosis (*k* = 4, SMD = -0.31) versus those including subthreshold features (*k* = 4, SMD = -0.24) revealed no significant difference (*Q* = 0.42, *p* = .52), though statistical power for this comparison was limited.

### Follow-up outcomes

Five studies (*k* = 5) reported follow-up data beyond post-treatment assessment, with follow-up periods ranging from 6 to 36 months. For these analyses, BPD symptom severity at follow-up was operationalized using the same instrument administered at post-treatment within each trial: BSL-23 (6), BPFSC (10, 11), ZAN-BPD (16), and the SCID-II BPD dimensional score (7). Where multiple follow-up timepoints were available, the longest available timepoint was used for the principal follow-up estimate, with intermediate timepoints reported separately. At 6-month follow-up (*k* = 3), treatment effects on BPD symptoms were maintained (SMD = -0.24, 95% CI: -0.48 to -0.01). Follow-up data from the MBT-G trial (40) showed partial maintenance of gains at 3 and 12 months. However, longer-term data from the Mohajerin et al. (16) trial showed progressive erosion of treatment gains: remission rates decreased from 45% at post-treatment to 28% at 12 months and approached control group levels (12% vs. 8%) at 36 months. The Mehlum et al. (6) 3-year follow-up similarly showed convergence between DBT-A and control groups over time. These findings suggest that treatment effects, while initially beneficial, may not persist without maintenance intervention.

## Discussion

### Summary of main findings

This systematic review and meta-analysis synthesized evidence from 12 randomized controlled trials with 844 participants. The results showed a mixed pattern of treatment effects: psychological interventions produced statistically significant improvements in BPD symptom severity (SMD = -0.27, *p* = .012), emotion regulation difficulties (SMD = -0.26, *p* = .020), and general psychopathology (SMD = -0.34, *p* = .003). All observed effects were small in magnitude according to conventional benchmarks (41). No significant effects were detected for depressive symptoms (SMD = -0.13, *p* = .24) or quality of life (SMD = 0.05, *p* = .80), suggesting that current interventions have limited impact on these domains.

Heterogeneity was consistently low across all outcomes (I^2^ = 0-29%), indicating that treatment effects were relatively homogeneous across studies despite variations in intervention types, treatment durations, and control conditions. The absence of significant publication bias, as evidenced by non-significant Egger’s tests across all outcomes (*p* >.15), further supports the robustness and reliability of these pooled estimates.

### Comparison with the adult BPD literature

The magnitude of effects observed here can be benchmarked against the more developed adult literature. Three recent syntheses (26–28) converge on a small-to-moderate pooled effect of structured psychotherapy in adults, with the largest meta-analysis estimating g around 0.32 on borderline-relevant outcomes. The present adolescent estimate for BPD symptom severity (SMD = −0.27) is broadly consistent with this adult pattern. Three observations align between the literatures. Effects across modalities are similarly modest, and head-to-head comparisons rarely identify a clearly superior intervention. Effects on broader outcomes (depression, quality of life, functioning) are smaller and less consistent than effects on BPD-specific symptoms in both age groups. Attenuation over follow-up is observed in both, raising parallel questions about maintenance. The adolescent literature diverges in two respects: fewer trials reach adequate statistical power, and comparator conditions are more heterogeneous, with several trials using non-manualized treatment-as-usual.

### Intervention-specific effects

In exploratory subgroup analyses, DBT-A showed the largest point estimate for effect on BPD symptoms (SMD = -0.71); however, this finding is based on a single trial (6, *n* = 77) and should not be interpreted as evidence of differential efficacy. Beyond the single-study limitation, the Mehlum trial employed an enhanced usual care control condition that may have been less active than controls in other studies, potentially inflating the between-group difference. The UP-A demonstrated promising effects (SMD = -0.45) in one study, suggesting that briefer transdiagnostic approaches deserve further study. The direct comparison between UP-A (12 sessions over 12 weeks) and MBT-A (12 months) in the Mohajerin et al. (16) trial revealed comparable post-treatment effects, which calls into question whether extended specialized treatments are necessary when briefer alternatives may achieve similar outcomes. These findings are broadly consistent with meta-analyses of psychological treatments for adult BPD, which have reported comparable small-to-moderate effect sizes (27, 42), though direct developmental comparisons are complicated by differences in outcome measures and follow-up periods. Subgroup analyses for secondary outcomes (**Supplementary Figures 1**-**S3**) revealed a similar pattern: for depressive symptoms, only DBT-A showed a trend toward significance (SMD = −0.42), while TFP-A showed no effect; for general psychopathology, PDT demonstrated the largest subgroup effect (SMD = −0.59); and for emotion regulation, ERT + TAU carried the largest weight but did not reach significance as a subgroup.

### Long-term outcomes

The trajectory of treatment effects over time is a critical consideration for clinical practice. Data from the Mohajerin et al. (16) trial showed progressive erosion of treatment gains: remission rates declined from 45% at post-treatment to 28% at 12-month follow-up, and approached control group levels (12% vs. 8%) at 36 months. The 3-year follow-up data from Mehlum et al. (6) similarly showed convergence between DBT-A and control conditions over time. These findings suggest that adolescent BPD may need to be conceptualized as a chronic condition requiring ongoing management rather than a discrete episode amenable to time-limited intervention. Whether maintenance treatments, booster sessions, or stepped-care approaches can prevent this erosion of gains remains an open question that future trials should directly address.

### Differential effects across outcome domains

The differential pattern of effects across outcome domains—reliable improvement on BPD-specific symptoms and general psychopathology, but null findings for depressive symptoms and quality of life—is relevant to understanding both the mechanisms and the practical limits of current treatments. One likely contributor is measurement specificity. BPD-specific instruments capture emotion dysregulation and impulsivity—the direct targets of most included interventions—whereas depression measures primarily assess anhedonia and hopelessness, which are not explicitly addressed in BPD-focused protocols. Quality of life, in turn, depends on functional domains such as peer relationships, academic performance, and family functioning that require sustained environmental change rather than symptom reduction alone. Developmental factors may also play a role: adolescent depression differs phenomenologically from adult depression, and standard depression scales may not capture the kinds of affective changes that occur during treatment. Similarly, quality of life in adolescence is heavily shaped by school and social contexts that most interventions do not directly target.

The high rate of comorbid depression in this population (~43% across studies numerically reporting these data; see Results) adds further complexity. BPD-focused interventions may not adequately address core depressive pathology, which would argue for integrating depression-specific components such as cognitive restructuring and behavioral activation. Treatment duration is another consideration: most included interventions lasted 16 weeks or less, which may be sufficient to reduce acute emotional instability but too short to produce improvements in quality of life, a domain that typically requires months of sustained environmental and functional change. At the level of therapeutic mechanism, these interventions appear to be effective at modifying emotional reactivity and impulsive behavior but may leave relatively untouched the persistent negative cognitions, anhedonia, and adverse life circumstances that maintain depressive symptoms.

### Clinical significance of treatment effects

The statistically significant but small treatment effects observed in this meta-analysis (SMD = 0.27-0.34 for significant outcomes) require careful interpretation regarding their clinical significance and practical implications for patient care.

#### Magnitude of change in clinical terms

The observed effect size of SMD = -0.27 for BPD symptom severity translates to approximately a 3.5-point reduction on the BSL-23 (*SD* ≈ 13), a 4.2-point reduction on the BPFSC (*SD* ≈ 15.5), and a 0.8-point reduction on the SCID-II BPD dimensional score (*SD* ≈ 3.0).

The established minimal clinically important difference (MCID) for BSL-23 in adults is 7.5 points (43). Our observed effect of ~3.5 points represents less than half the adult MCID threshold. No validated MCID exists for adolescent BPD measures, but if adolescent thresholds are comparable, our effects would fall below the level patients typically notice as meaningful improvement.

Several caveats apply, however. A 3.5-point reduction in moderate-to-severe symptoms represents approximately 15–20% symptom reduction, which may be clinically valuable in a severe, chronic disorder even if it falls below the formal MCID. The MCID is also a probabilistic threshold, not a hard cutoff: effects below it still benefit some individuals, and our SMD of -0.27 suggests that approximately 35–40% of treated patients improve more than the average control patient. For a disorder with 70–80% self-harm rates, even modest symptom reductions might meaningfully lower the risk of acute self-injurious behavior.

#### Clinical significance conclusion

On balance, the statistical significance of our findings is clear: treatment effects are reliable, replicated across studies with low heterogeneity, and unlikely attributable to chance. Whether these effects are clinically meaningful is less certain. The modest magnitude of observed effects, falling below established MCID thresholds, suggests that while statistically detectable, benefits may be clinically marginal for a substantial proportion of patients. We would still recommend offering psychological interventions to adolescents with BPD, as they provide small but real benefits for a severe condition with limited treatment alternatives. At the same time, clinicians should set realistic expectations with patients and families and monitor outcomes across multiple domains. Non-responders may require intensification or modification of treatment, and the evidence of effect erosion over time argues for extended treatment durations. Comorbid conditions, particularly depression, may need to be addressed through adjunctive or sequential intervention.

### Clinical implications

These findings have practical relevance for treatment planning. Regarding treatment selection, DBT-A and UP-A showed the most promising effects in the current evidence base, though both rest on limited data. The comparable efficacy of UP-A (a 12-week transdiagnostic protocol) and MBT-A (a 12-month specialized treatment) raises the possibility that briefer approaches may offer similar benefits with greater efficiency and accessibility—a finding particularly relevant for resource-limited settings. Clinicians should communicate realistic expectations to adolescents and their families; our findings indicate that psychological interventions produce modest symptomatic improvements on average, and substantial residual symptoms are likely to persist. Framing treatment as one component of a longer-term management strategy may help prevent discouragement and premature discontinuation. The differential effects across outcome domains also highlight the need for multi-domain outcome monitoring: improvement in BPD-specific symptoms should not be assumed to generalize to depression or quality of life, and routine assessment across domains can inform decisions about adjunctive interventions. Quality of life gains, in particular, may depend on broader support beyond the index intervention, including family therapy and school consultation; with respect to treatment duration, brief interventions of 12–16 weeks may be adequate for acute symptom management, but functional recovery likely requires 6–12 months or longer. The erosion of treatment gains observed in follow-up studies suggests that brief, time-limited interventions may be insufficient for durable improvement; clinicians should consider extended treatment durations, planned booster sessions, or maintenance phases. The null findings for depressive symptoms, despite high comorbidity rates (~43% across studies numerically reporting these data), further suggest that BPD-focused interventions may not adequately address co-occurring depression, and integration of depression-specific components or sequential treatment deserves consideration.

### Limitations

Several methodological limitations should be considered when interpreting these findings. The modest number of studies per outcome (*k* = 2–8) limited statistical power for subgroup and moderator analyses, and findings regarding differential efficacy across intervention types should therefore be regarded as exploratory rather than definitive. Relatedly, all included trials were open-label—unavoidable in psychotherapy research—which introduces potential for performance and detection bias. The predominant reliance on self-report measures may have amplified expectancy effects; future trials incorporating clinician-rated or behavioral outcomes could yield more objective efficacy estimates. The demographic composition of included samples also limits generalizability. The predominance of female participants (81%), while reflecting clinical epidemiology, leaves treatment efficacy in male adolescents poorly characterized, and the concentration of studies in Western, high-income countries raises questions about cross-cultural applicability. Heterogeneity in control conditions is a further concern: comparisons ranged from waitlist to treatment-as-usual to active comparators, and trials using less active controls likely overestimate true treatment effects. Most studies also had relatively short follow-up periods (≤12 months), precluding definitive conclusions about long-term durability, though the available longer-term data point to concerning erosion of gains. Finally, despite the use of robust variance estimation, residual statistical dependencies from multiple effect sizes within studies cannot be ruled out.

Beyond methodological heterogeneity in the included trials, our synthesis also reflects an underlying conceptual heterogeneity in how BPD is defined in adolescence. Included trials operationalized BPD in different ways, ranging from full DSM-IV/5 BPD diagnoses via structured interview to subthreshold criterion counts or cutoff-based screening, and the populations described by these different operationalizations are not perfectly equivalent. This conceptual heterogeneity, compounded by the universally high rate of psychiatric comorbidity in adolescent BPD samples, places a fundamental limit on the extent to which any meta-analytic synthesis (including ours) can characterize “treatment efficacy for BPD” as a single unitary construct. Future trials and reviews will need to address this through clearer operationalization and reporting standards.

### Future research directions

Several directions for future research follow from this review. The most pressing need is for adequately powered head-to-head trials directly comparing active treatments (e.g., DBT-A vs. MBT-A vs. UP-A), as current evidence does not permit reliable conclusions about differential efficacy. Equally important is extending follow-up periods to two years or longer, with systematic assessment of maintenance interventions, to clarify the durability of treatment effects and optimal continuation strategies. Research on predictors and moderators of treatment response—including baseline severity, comorbidity profiles, attachment patterns, and family factors—would enable more personalized treatment matching. Given the null findings for depression and quality of life, future trials should also prioritize these outcomes and test whether augmentation with disorder-specific components improves broader functioning. The evidence base would benefit from greater cultural and geographic diversity, as the current concentration in Western, high-income countries limits generalizability. On the measurement side, developing and validating minimal clinically important difference thresholds for adolescent BPD measures would allow more meaningful interpretation of treatment effects. Mechanistic studies examining putative mediators—such as emotion regulation capacity, mentalization, and interpersonal functioning—could further inform the refinement of existing interventions.

## Conclusions

In conclusion, psychological interventions for adolescent BPD are associated with small but statistically significant improvements in BPD symptom severity, emotion regulation, and general psychopathology, with effects that are consistent across studies and robust to sensitivity analyses. However, no significant effects were observed for depressive symptoms or quality of life, and available long-term data suggest that treatment gains may attenuate over time without ongoing intervention. Current psychological treatments should therefore be viewed as beneficial but limited tools: realistic expectations, routine multi-domain outcome monitoring, and planning for extended or maintenance treatment are all important. Adequately powered head-to-head trials comparing active treatments and longer follow-up periods examining maintenance strategies are needed. Future work should also identify predictors of treatment response, develop interventions with broader impact across outcome domains, and establish validated clinical significance thresholds for adolescent BPD measures.

## Data Availability

The original contributions presented in the study are included in the article/**Supplementary Material**. Further inquiries can be directed to the corresponding author.
