# Supplementary material for: Efficacy of psychological interventions for adolescents with borderline personality disorder: a systematic review and meta-analysis
Source: Front Psychiatry. 2026 Jun 11;17:1833555. doi: 10.3389/fpsyt.2026.1833555 (PMC13294224; doi:10.3389/fpsyt.2026.1833555)
Supplement: Supplementary file 1 [file Table1.docx]

**Supplementary Material**

**Efficacy of Psychological Interventions for Adolescents with Borderline Personality Disorder:**

**A Systematic Review and Meta-Analysis**

*PROSPERO Registration: CRD420261306339*

# **Supplementary Table S1. PRISMA 2020 Checklist**

| **Section and Topic** | **#** | **Checklist Item** | **Location** |
| --- | --- | --- | --- |
| **TITLE** | | | |
| **Title** | 1 | Identify the report as a systematic review. | Title page |
| **ABSTRACT** | | | |
| **Abstract** | 2 | See the PRISMA 2020 for Abstracts checklist. | Abstract |
| **INTRODUCTION** | | | |
| **Rationale** | 3 | Describe the rationale for the review in the context of existing knowledge. | Introduction, para 1–2 |
| **Objectives** | 4 | Provide an explicit statement of the objective(s) or question(s) the review addresses. | Introduction, Study Aims |
| **METHODS** | | | |
| **Eligibility criteria** | 5 | Specify the inclusion and exclusion criteria for the review and how studies were grouped for the syntheses. | Methods, Eligibility Criteria |
| **Information sources** | 6 | Specify all databases, registers, websites, organisations, reference lists and other sources searched or consulted to identify studies. | Methods, Search Strategy |
| **Search strategy** | 7 | Present the full search strategies for all databases, registers and websites, including any filters and limits used. | Supplementary Table S2 |
| **Selection process** | 8 | Specify the methods used to decide whether a study met the inclusion criteria of the review. | Methods, Eligibility Criteria |
| **Data collection process** | 9 | Specify the methods used to collect data from reports, including how many reviewers collected data, whether they worked independently, and any processes for obtaining or confirming data. | Methods, Data Extraction |
| **Data items** | 10a | List and define all outcomes for which data were sought. Specify whether all results that were compatible with each outcome domain in each study were sought. | Methods, Data Extraction |
|  | 10b | List and define all other variables for which data were sought. | Methods, Data Extraction |
| **Study risk of bias assessment** | 11 | Specify the methods used to assess risk of bias in the included studies. | Methods; Results, Risk of Bias |
| **Effect measures** | 12 | Specify for each outcome the effect measure(s) used in the synthesis or presentation of results. | Methods, Effect Size Calculation |
| **Synthesis methods** | 13a | Describe the processes used to decide which studies were eligible for each synthesis. | Methods, Meta-Analytic Procedures |
|  | 13b | Describe any methods required to prepare the data for presentation or synthesis. | Methods, Effect Size Calculation |
|  | 13c | Describe any methods used to tabulate or visually display results of individual studies and syntheses. | Results, Figures 3–11 |
|  | 13d | Describe any methods used to synthesize results and provide a rationale for the choice(s). | Methods, Meta-Analytic Procedures |
|  | 13e | Describe any methods used to explore possible causes of heterogeneity among study results. | Methods, Sensitivity Analyses |
|  | 13f | Describe any sensitivity analyses conducted to assess robustness of the synthesized results. | Methods, Sensitivity Analyses |
| **Reporting bias assessment** | 14 | Describe any methods used to assess risk of bias due to missing results in a synthesis. | Methods, Publication Bias |
| **Certainty assessment** | 15 | Describe any methods used to assess certainty in the body of evidence for an outcome. | N/A |
| **RESULTS** | | | |
| **Study selection** | 16a | Describe the results of the search and selection process. | Results, Study Selection; Figure 1 |
|  | 16b | Cite studies that might appear to meet the inclusion criteria, but which were excluded, and explain why. | Figure 1 (PRISMA flow) |
| **Study characteristics** | 17 | Cite each included study and present its characteristics. | Results, Table 1 |
| **Risk of bias in studies** | 18 | Present assessments of risk of bias for each included study. | Results, Figure 12 |
| **Results of individual studies** | 19 | For all outcomes, present for each study: (a) summary statistics for each group and (b) an effect estimate and its precision. | Results, Figures 3–11 |
| **Results of syntheses** | 20a | For each synthesis, briefly summarise the characteristics and risk of bias among contributing studies. | Results sections |
|  | 20b | Present results of all statistical syntheses conducted. | Results; Table 2 |
|  | 20c | Present results of all investigations of possible causes of heterogeneity. | Results, Sensitivity Analyses; Supplementary Figures S1–S3 |
|  | 20d | Present results of all sensitivity analyses conducted. | Results, Sensitivity Analyses |
| **Reporting biases** | 21 | Present assessments of risk of bias due to missing results for each synthesis assessed. | Results, Funnel Plots; Egger’s test |
| **Certainty of evidence** | 22 | Present assessments of certainty in the body of evidence for each outcome assessed. | Discussion, Clinical Significance |
| **DISCUSSION** | | | |
| **Discussion** | 23a | Provide a general interpretation of the results in the context of other evidence. | Discussion |
|  | 23b | Discuss any limitations of the evidence included in the review. | Discussion, Limitations |
|  | 23c | Discuss any limitations of the review processes used. | Discussion, Limitations |
|  | 23d | Discuss implications of the results for practice, policy, and future research. | Discussion, Clinical Implications; Future Research |
| **OTHER INFORMATION** | | | |
| **Registration and protocol** | 24a | Provide registration information for the review, including register name and registration number. | Methods (PROSPERO: CRD420261306339) |
|  | 24b | Indicate where the review protocol can be accessed, or state that a protocol was not prepared. | Methods |
|  | 24c | Describe and explain any amendments to information provided at registration or in the protocol. | N/A – no amendments |
| **Support** | 25 | Describe sources of financial or non-financial support for the review. | Funding |
| **Competing interests** | 26 | Declare any competing interests of review authors. | Conflicts of Interest |
| **Availability of data, code and other materials** | 27 | Report which of the following are publicly available and where they can be found: template data collection forms; data extracted from included studies; data used for all analyses; analytic code; any other materials used in the review. | Data Availability Statement |

*Note. From Page et al. (2021). The PRISMA 2020 statement: an updated guideline for reporting systematic reviews. BMJ, 372, n71.*

# **Supplementary Table S2. Complete Search Strategy**

**PubMed/MEDLINE**

Search: ((((("borderline personality disorder"[MeSH Terms]) OR ("borderline personality"[tiab] OR "BPD"[tiab] OR "emotionally unstable personality"[tiab] OR "personality disorder"[tiab]))) AND ((("adolescent"[MeSH Terms]) OR ("adolescen*"[tiab] OR "teen*"[tiab] OR "youth*"[tiab] OR "young people"[tiab] OR "juvenile"[tiab] OR "minor*"[tiab] OR "pediatric"[tiab] OR "paediatric"[tiab])))) AND ((("psychotherapy"[MeSH Terms] OR "cognitive behavioral therapy"[MeSH Terms]) OR ("psychotherap*"[tiab] OR "cognitive behavio*"[tiab] OR "dialectical behavio*"[tiab] OR "DBT"[tiab] OR "mentali*"[tiab] OR "MBT"[tiab] OR "cognitive analytic"[tiab] OR "CAT"[tiab] OR "emotion regulat*"[tiab] OR "schema therap*"[tiab] OR "transference focused"[tiab] OR "unified protocol"[tiab] OR "psychological intervention*"[tiab] OR "psychological treatment*"[tiab])))) AND (("randomized controlled trial"[pt] OR "controlled clinical trial"[pt] OR "randomized"[tiab] OR "randomised"[tiab] OR "placebo"[tiab] OR "trial"[tiab] OR "randomly"[tiab]) NOT ("animals"[MeSH] NOT "humans"[MeSH])) Filters: Inception to January 10, 2026; No language restrictions

**PsycINFO (via Ovid)**

1 exp borderline personality disorder/ 2 (borderline personality or BPD or emotionally unstable personality).ti,ab. 3 1 or 2 4 exp adolescent/ 5 (adolescen* or teen* or youth* or young people or juvenile or minor* or pediatric or paediatric).ti,ab. 6 4 or 5 7 exp psychotherapy/ or exp cognitive behavior therapy/ 8 (psychotherap* or cognitive behavio* or dialectical behavio* or DBT or mentali* or MBT or cognitive analytic or CAT or emotion regulat* or schema therap* or transference focused or unified protocol or psychological intervention* or psychological treatment*).ti,ab. 9 7 or 8 10 (randomized or randomised or randomly or trial or RCT or controlled trial or placebo).ti,ab. 11 3 and 6 and 9 and 10 12 limit 11 to yr="1980-2026"

**Embase (via Ovid)**

#1 ‘borderline personality disorder’/exp #2 borderline:ab,ti AND personality:ab,ti OR ‘BPD’:ab,ti OR ‘emotionally unstable personality’:ab,ti #3 #1 OR #2 #4 ‘adolescent’/exp #5 adolescen*:ab,ti OR teen*:ab,ti OR youth*:ab,ti OR ‘young people’:ab,ti OR juvenile:ab,ti OR pediatric:ab,ti OR paediatric:ab,ti #6 #4 OR #5 #7 ‘psychotherapy’/exp OR ‘cognitive behavioral therapy’/exp #8 psychotherap*:ab,ti OR ‘cognitive behavio*’:ab,ti OR ‘dialectical behavio*’:ab,ti OR ‘DBT’:ab,ti OR mentali*:ab,ti OR ‘MBT’:ab,ti OR ‘cognitive analytic’:ab,ti OR ‘emotion regulat*’:ab,ti OR ‘schema therap*’:ab,ti OR ‘transference focused’:ab,ti OR ‘unified protocol’:ab,ti OR ‘psychological intervention*’:ab,ti OR ‘psychological treatment*’:ab,ti #9 #7 OR #8 #10 ‘randomized controlled trial’/exp OR ‘crossover procedure’/exp OR ‘double blind procedure’/exp OR ‘single blind procedure’/exp #11 random* OR factorial* OR crossover* OR placebo* OR assign* OR allocat* OR volunteer* #12 #10 OR #11 #13 #3 AND #6 AND #9 AND #12

**Cochrane CENTRAL**

#1 MeSH descriptor: [Borderline Personality Disorder] explode all trees #2 (borderline personality OR BPD OR "emotionally unstable personality"):ti,ab,kw (Word variations have been searched) #3 #1 OR #2 #4 MeSH descriptor: [Adolescent] explode all trees #5 (adolescen* OR teen* OR youth* OR "young people" OR juvenile OR pediatric OR paediatric):ti,ab,kw (Word variations have been searched) #6 #4 OR #5 #7 MeSH descriptor: [Psychotherapy] explode all trees #8 MeSH descriptor: [Cognitive Behavioral Therapy] explode all trees #9 (psychotherap* OR "cognitive behavio*" OR "dialectical behavio*" OR DBT OR mentali* OR MBT OR "cognitive analytic" OR CAT OR "emotion regulat*" OR "schema therap*" OR "transference focused" OR "unified protocol" OR "psychological intervention*" OR "psychological treatment*"):ti,ab,kw (Word variations have been searched) #10 #7 OR #8 OR #9 #11 #3 AND #6 AND #10

**Grey Literature and Additional Sources**

*ProQuest Dissertations & Theses Global (search terms: borderline personality disorder AND adolescen* AND (psychotherapy OR DBT OR MBT OR cognitive analytic OR emotion regulation OR unified protocol) AND (randomized OR trial)); ISSPD conference proceedings (2015–2025); reference lists of all included studies and prior systematic reviews. No language restrictions were applied across all databases.*

# **Supplementary Figures: Subgroup Analyses by Intervention Type**

**Supplementary Figure S1.** Subgroup Analysis by Intervention Type: Effect on Depressive Symptom Severity


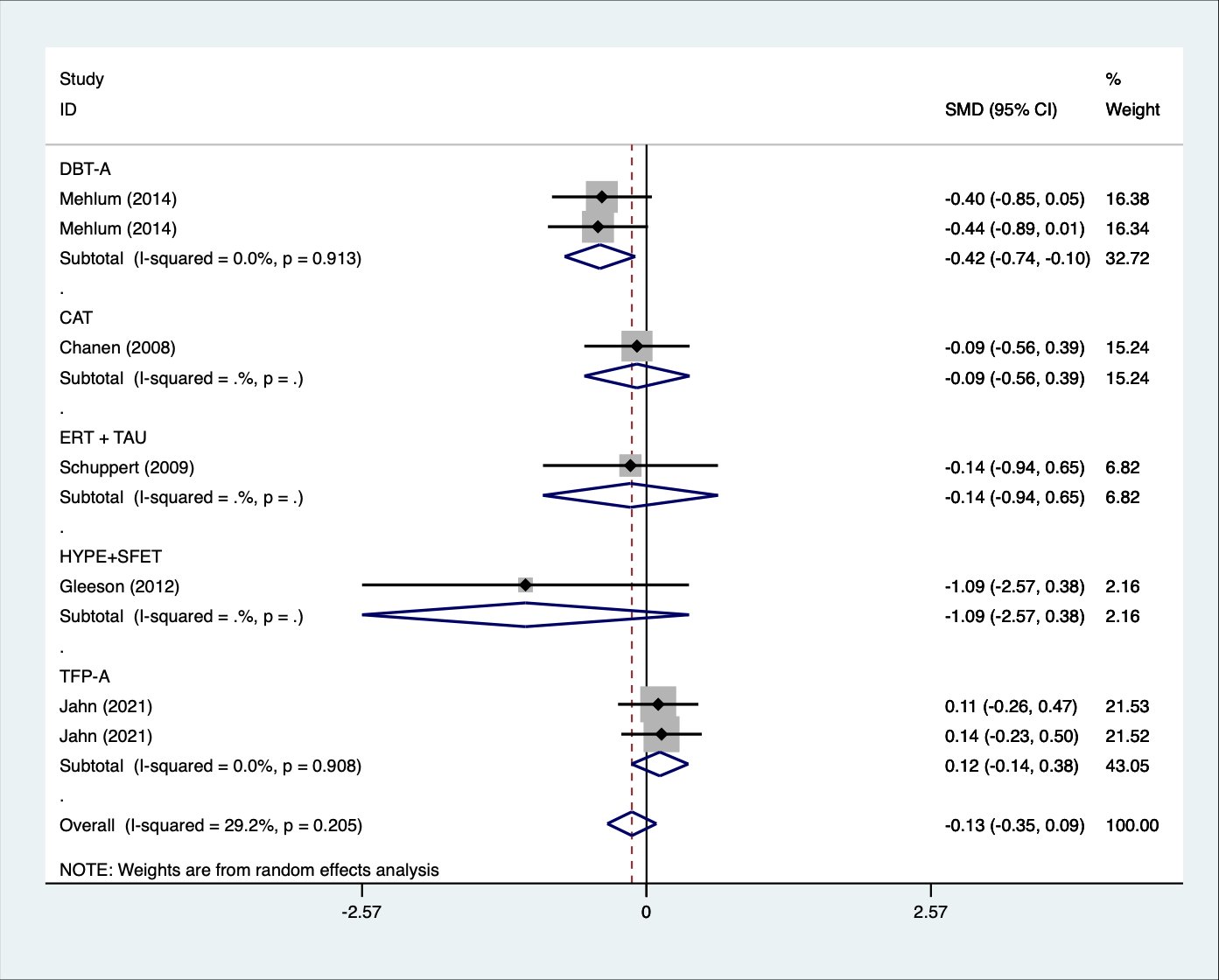


***Note.*** *Forest plot showing subgroup analysis by intervention type for depressive symptom severity. Subgroups: DBT-A (k = 2, SMD = −0.42), CAT (k = 1, SMD = −0.09), ERT + TAU (k = 1, SMD = −0.14), HYPE+SFET (k = 1, SMD = −1.09), TFP-A (k = 2, SMD = 0.12). Overall: SMD = −0.13 (95% CI: −0.35 to 0.09), I² = 29.2%. Random-effects model.*

**Supplementary Figure S2.** Subgroup Analysis by Intervention Type: Effect on Emotion Regulation Difficulties


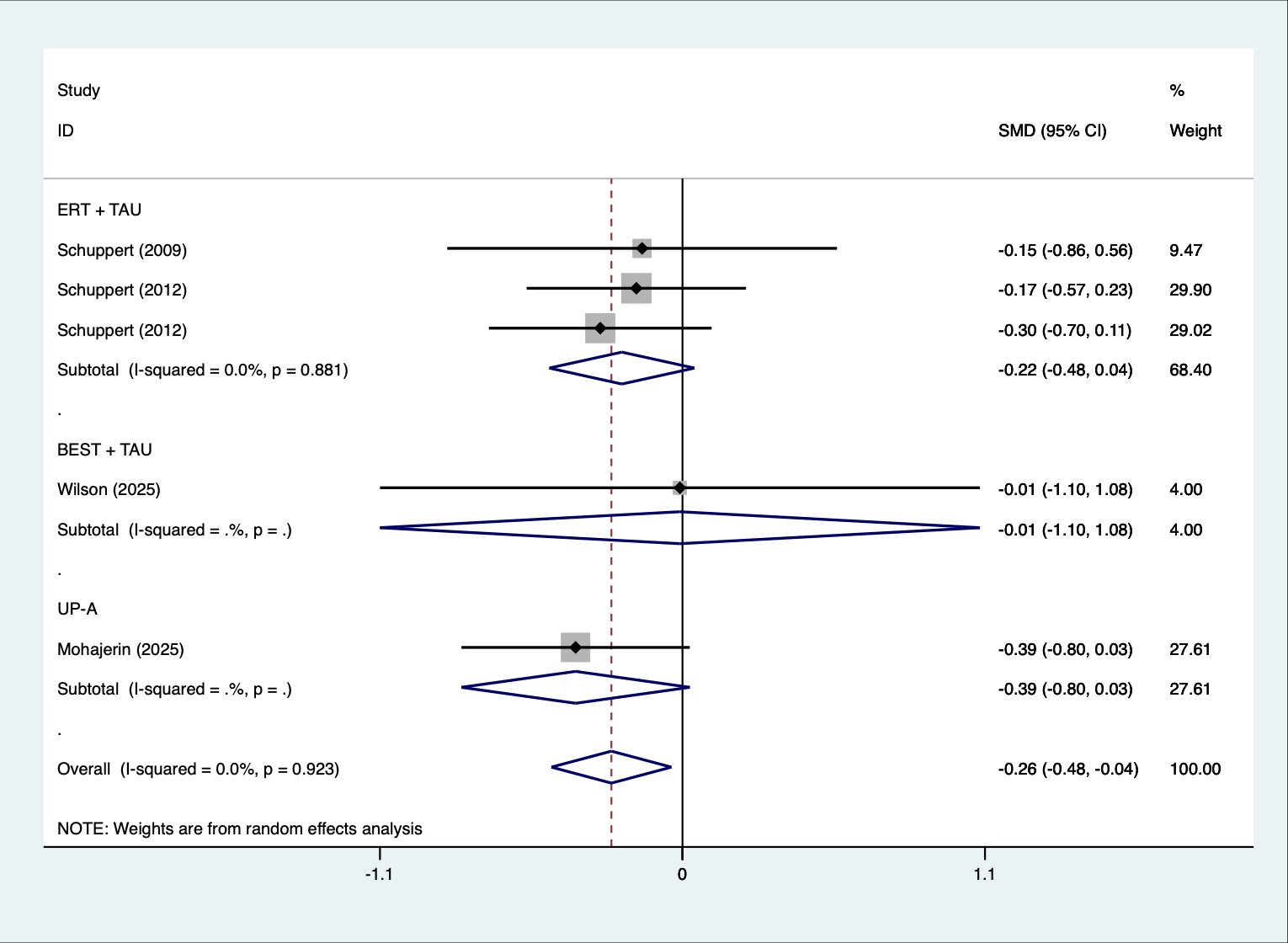


***Note.*** *Forest plot showing subgroup analysis by intervention type for emotion regulation difficulties. Subgroups: ERT + TAU (k = 3, SMD = −0.22, weight = 68.40%), BEST + TAU (k = 1, SMD = −0.01), UP-A (k = 1, SMD = −0.39). Overall: SMD = −0.26 (95% CI: −0.48 to −0.04), I² = 0.0%. Random-effects model.*

**Supplementary Figure S3.** Subgroup Analysis by Intervention Type: Effect on General Psychopathology


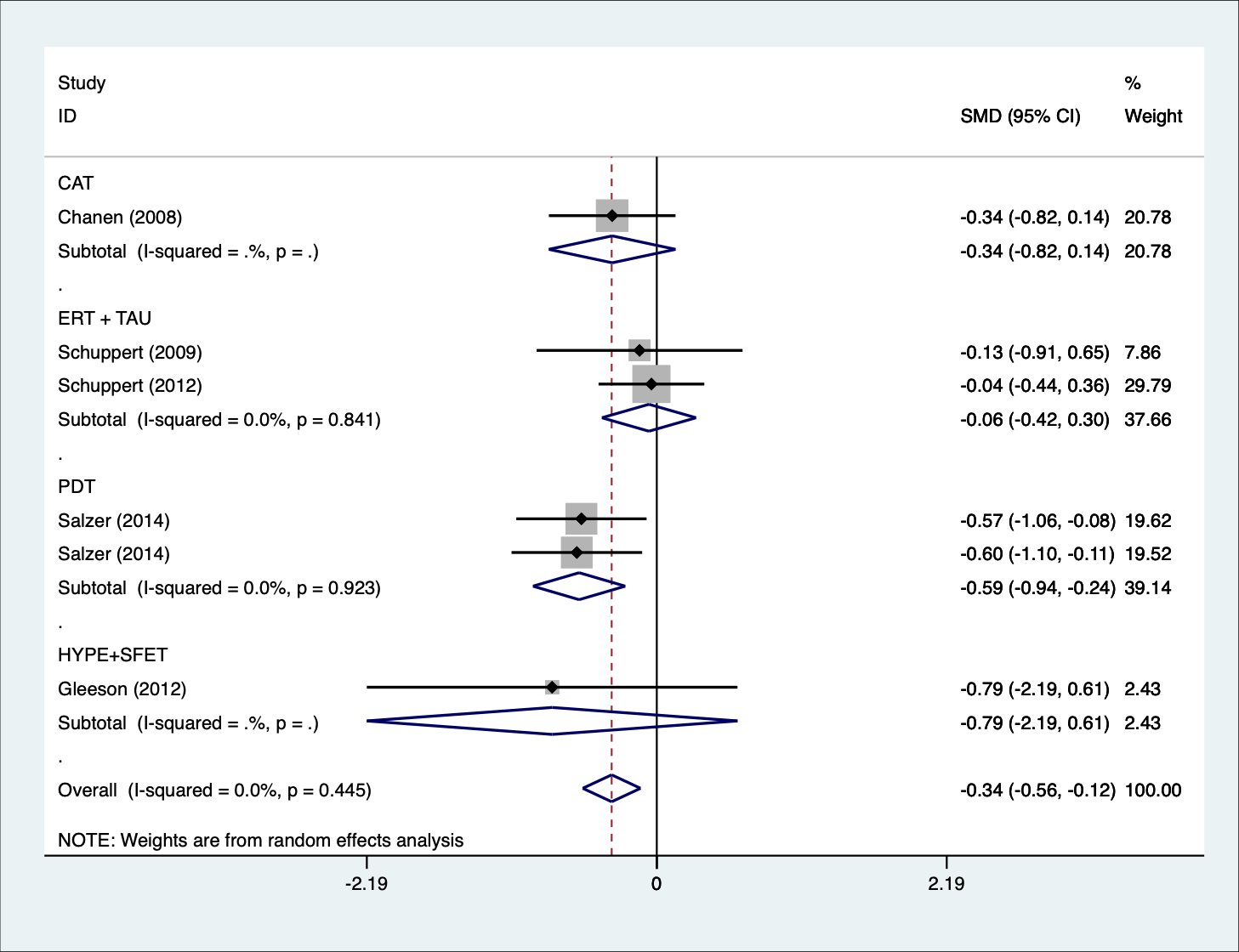


***Note.*** *Forest plot showing subgroup analysis by intervention type for general psychopathology. Subgroups: CAT (k = 1, SMD = −0.34), ERT + TAU (k = 2, SMD = −0.06), PDT (k = 2, SMD = −0.59, weight = 39.14%), HYPE+SFET (k = 1, SMD = −0.79). Overall: SMD = −0.34 (95% CI: −0.56 to −0.12), I² = 0.0%. PDT showed the largest significant subgroup effect. Random-effects model.*
